# Quantifying the Single‐Cell Morphological Landscape of Cellular Transdifferentiation through Force Field Reconstruction

**DOI:** 10.1002/advs.202512325

**Published:** 2025-11-07

**Authors:** Chudan Yu, Chuanbo Liu, Erkang Wang, Jin Wang

**Affiliations:** ^1^ College of Chemistry Jilin University Changchun Jilin 130012 P. R. China; ^2^ State Key Laboratory of Electroanalytical Chemistry Changchun Institute of Applied Chemistry Chinese Academy of Sciences Changchun Jilin 130022 P. R. China; ^3^ Department of Chemistry Department of Physics and Astronomy State University of New York Stony Brook NY 11794‐3400 USA

**Keywords:** force field reconstruction, morphological change, transdifferentiation

## Abstract

Advancements in sequencing technologies have reshaped our understanding of cell behaviors through transcriptomics, but a gap remains in developing quantitative models for multi‐omic data, especially for cellular morphology changes. A pivotal challenge is the lack of cell‐specific velocity information, crucial for reconstructing global velocity fields to analyze dynamics and thermodynamics in cellular process. In this study, fibroblast‐to‐neuron transdifferentiation snapshots are captured and a novel machine learning approach to reconstruct the underlying force field of the morphological change from the sparse sampled single‐cell imaging data is developed. The methodology involves decomposing the driving force field into a flow flux force field and a gradient of a time‐dependent potential, extending the landscape and flux framework to non‐steady‐state conditions. This study demonstrates that the reconstructed force field accurately captures the intrinsic morphological landscape of cell fate switching and reveals the impact of noise on state transitions. This approach offers a general framework for analyzing single‐cell morphological data and holds promise for application to other single‐cell multi‐omic datasets lacking inherent velocity information.

## Graphical abstract

This study reconstructs the driving force field of fibroblast‐to‐neuron transdifferentiation from sparse single‐cell images by decomposing it into flux and time‐dependent potential gradient, extending the landscape‐flux framework to non‐steady‐state systems. This approach accurately captures the morphological landscape of cell fate switching, reveals noise's role in transitions, and provides a general framework for analyzing single‐cell data lacking velocity information.

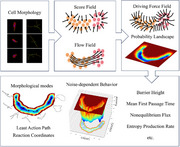

## 1 Introduction

Cell transdifferentiation represents a process whereby a terminally differentiated cell alters its lineage, adopting the distinct morphology and function characteristic of a different cell type.^[^ ^1^ ^]^ This paradigm shifts the traditional notions of developmental biology, which assert that cellular fate is irreversibly determined along specific differentiation pathways.^[^ ^2^ ^]^ The implications of transdifferentiation are significant for regenerative medicine, disease modeling, and the broader understanding of cellular plasticity. Unlike processes involving pluripotent cells, direct lineage conversion is proposed to offer a safer and more efficient pathway for therapeutic applications, circumventing the teratoma formation risk and largely preserving donor‐specific signatures.^[^ ^3^, ^4^ ^]^ This phenotypic transformation entails comprehensive changes in gene expression, cellular structure, and function, which are pivotal for dissecting the mechanisms and expanding the potential applications of this phenomenon.^[^ ^5^ ^]^

Recent advancements in single‐cell RNA sequencing (scRNA‐seq) have substantially elucidated the alterations in genetic and epigenetic landscapes during the process of direct cell reprogramming.^[^ ^3^ ^]^ Nonetheless, the influence of morphological changes, particularly those mediated by cytoskeletal architecture regulation, on reprogramming dynamics remains poorly characterized. Cellular morphology fundamentally emerges from the intricate interplay of comprehensive molecular networks that meticulously govern cellular behaviors. Morphological adaptations reflect cellular responses to external perturbations and stressors, thus offering an additional dimension to the profiling of cell reprogramming.^[^ ^6^ ^]^ The integration of morphological data with genomic, transcriptomic, proteomic, and metabolomic datasets promises to furnish a holistic perspective of cellular function and regulation. In multi‐omic analyses, morphological transformations can be correlated with distinct molecular signatures, facilitating the identification of biomarkers relevant to disease states or therapeutic responses. For instance, cancer cells frequently exhibit unique morphological traits associated with specific genetic mutations or expression profiles. Examining these morphological variations concurrently with other single‐cell data sets can enhance our understanding of pivotal morphology‐related biological processes, such as tumor heterogeneity, thereby unveiling potential therapeutic targets. The incorporation of morphological data into multi‐omic analyses offers a comprehensive perspective on the biological systems involved, potentially enhancing the precision and efficacy of targeted medical intervention.^[^ ^7^ ^]^

However, quantitatively understanding the cell morphology change poses significant challenges due to the inherently irregular shapes and substantial variability among different cell types. Traditional geometric or statistical descriptors, such as area, aspect ratio, curvature, and roughness, are typically calculated to identify and measure the morphological features of individual cells. These descriptors facilitate the quantification of deviations in cell shapes and enable cross‐modality predictions.^[^ ^6^, ^8^, ^9^, ^10^ ^]^ While these geometric features are straightforward and maintain simplicity compared to more complex approaches, they fail to adequately capture morphological differences, as the distance in feature space does not necessarily reflect variations in cell shape.^[^ ^11^ ^]^ This underscores the limitations of handcrafted feature extractors in representing complex structures. A more robust approach involves utilizing the raw spatial coordinates of cell contours to discern morphological signatures via unsupervised learning method.^[^ ^11^, ^12^ ^]^ By extracting a sufficient number of equally spaced points from each cell contour, these coordinates are used to construct a high‐dimensional feature space that represents the intricate morphology of cells. Unsupervised learning techniques, such as clustering or dimensional reduction, can then be employed to analyze changes in cell morphology. The centroids of these single‐cell morphology clusters define the “shape modes” of cell contours, and projecting cellular dynamics onto a reduced dimensional space facilitates visualization of state distributions and evolutionary trajectories. However, given the invariance of cell morphology to rigid spatial transformations, it is crucial to correctly align the indices of cell morphology features. Conventional contour alignment procedures often fail to quantify morphological differences between highly dissimilar cells, especially in the case of branched neural cells. Consequently, a transformation‐invariant distance measure, such as the Gromov–Hausdorff distance, proves effective in quantifying morphological distances, even for complex neural cell structures.^[^ ^13^ ^]^ Machine learning methods are also employed to extract morphological features with variational autoencoders with transformation invariance.^[^ ^14^, ^15^ ^]^

Even with proper morphological features, deciphering the intrinsic dynamics governing changes in cell morphology remains a formidable challenge. Previous studies have leveraged established biological regulatory networks to model and reconstruct the underlying morphological landscape of cellular morphological transformations.^[^ ^16^ ^]^ However, the inherent simplification of these reduced regulatory network models may obscure the complexities of the intrinsic dynamics involved. Moreover, the estimation of numerous kinetic parameters is necessitated by the sparse nature of sampled data, which often leads to questionable accuracy. In practice, assumptions regarding trivial values for many of these parameters are made based on minimal prior knowledge. As a result, these investigations predominantly yield phenomenological insights into the data, while offering limited predictive capacity. The appropriate approach for elucidating the dynamics of morphological changes should directly quantify the process using experimental data obtained from single‐cell morphological assessments, such as microscopic imaging of cells.

In addressing these challenges, we introduce the sparse Force Field Reconstruction (sparseFFR) methodology, a comprehensive approach for deducing the underlying landscape and quantifying both dynamics and thermodynamics of systems from morphological single‐cell datasets. This method hinges on the decomposition of the driving force field into a flow flux field (hereafter referred to as the flow field) and a score field, which represents the gradient of a potential field. This decomposition extends the landscape and flux framework of nonequilibrium thermodynamics to encompass non‐steady‐state conditions. By concurrently estimating the flow and score fields, we can reconstruct a parameterized, smooth force field across the entire low‐dimensional embedding space through the application of sparse vector field consensus.^[^ ^17^ ^]^ To evaluate and substantiate our methodology, we conducted a series of experiments focused on the direct reprogramming of fibroblasts into neurons. Throughout the transdifferentiation process, alterations in cell morphology were recorded by snapshot imaging of fluorescent labeled fixed cells, and detailed morphological features at the single‐cell level were extracted from the imaging data. Subsequently, we deploy sparseFFR to elucidate the mechanisms underlying the transdifferentiation from the obtained data of fibroblasts to neurons. The reconstructed driving force field facilitates the recovery of Langevin dynamics governing cell morphological evolution from sparse sampled single‐cell morphological data, thereby enabling the generation of densely sampled trajectories. From this ensemble of densely sampled trajectories, we can infer the landscape using the steady‐state distribution, and quantify dynamics and thermodynamics such as barrier height, MFPT, and entropy production rate, thereby providing a thorough description of the underlying nonequilibrium dynamics and thermodynamics.

The recovered Langevin dynamics also facilitate the investigation of stochastic influences on morphological landscapes and phase transition mechanisms. Our study uncovers a two‐phase mechanism that emerges when noise is large enough to make the morphological landscape barriers shallower; this mechanism effectively explicates the morphological transformation observed during the transdifferentiation of fibroblasts into pre‐mature neural cells. The kinetic parameters governing this coarse‐grained two‐phase dynamics are fully delineated by the barrier height demarcating these two phases. An extensive analysis of gene expression regulation corroborates the two‐phase model, providing consistent evidence from both molecular and structural dimensions. Thermodynamic analyses further elucidate the dependence of thermodynamics on noise intensity, shedding light on energy conversion efficiency and the stability of nonequilibrium phases.

By mapping the morphological landscape of fibroblast‐to‐neuron conversion through sparseFFR, we gain profound insights into key morphological transitions, morphological shape distributions, and pinpointing pivotal stages of the process. The sparseFFR methodology also holds potential for examining mechanisms in other single‐cell datasets lacking inherent velocity information within direct cell reprogramming contexts, thereby enhancing our comprehension of cell fate regulation.

## 2 Results

### 2.1 Transdifferentiation of Human Fibroblasts to Neurons through Direct Reprogramming

A series of influential studies have elucidated the capability to generate functional neurons from both stem and somatic cell lineages through direct cell conversion. Evidence from several studies indicates that the application of neuronal‐specific transcription factors or small molecules enables the effective transdifferentiation of fibroblasts into functional neuron.^[^ ^18^, ^19^ ^]^ The fibroblast‐to‐neuron transdifferentiation process encompasses a sophisticated array of molecular events, encompassing neuron‐specific transcription factor expression, epigenetic reprogramming, and morphological transformation. During this conversion, fibroblasts exhibit neurite outgrowth, develop post‐synaptic densities, and exhibit electrical activity characteristic of neurons, reflecting both functional and morphological alterations. The cellular morphological transformation in fibroblast‐to‐neuron transdifferentiation is particularly intriguing, entailing cytoskeletal reorganization, alterations in cell adhesion properties, and the emergence of a polarized, neuron‐like morphology.^[^ ^18^ ^]^ This morphological evolution is fundamental to the cells’ neuronal functionality, as exemplified by neurite extension, which is crucial for synaptic connectivity and integration into neural networks. Elucidating the molecular mechanisms underpinning this morphological transformation is imperative for enhancing the efficiency and functionality of fibroblast‐to‐neuron conversion. In addition, this understanding offers significant insights into the broader concepts of cellular plasticity and the regulatory mechanisms governing cell morphology and function. Within this context, we utilize the fibroblast‐to‐neuron transdifferentiation process as a case study to explore the sparseFFR method.

**Figure 1:**
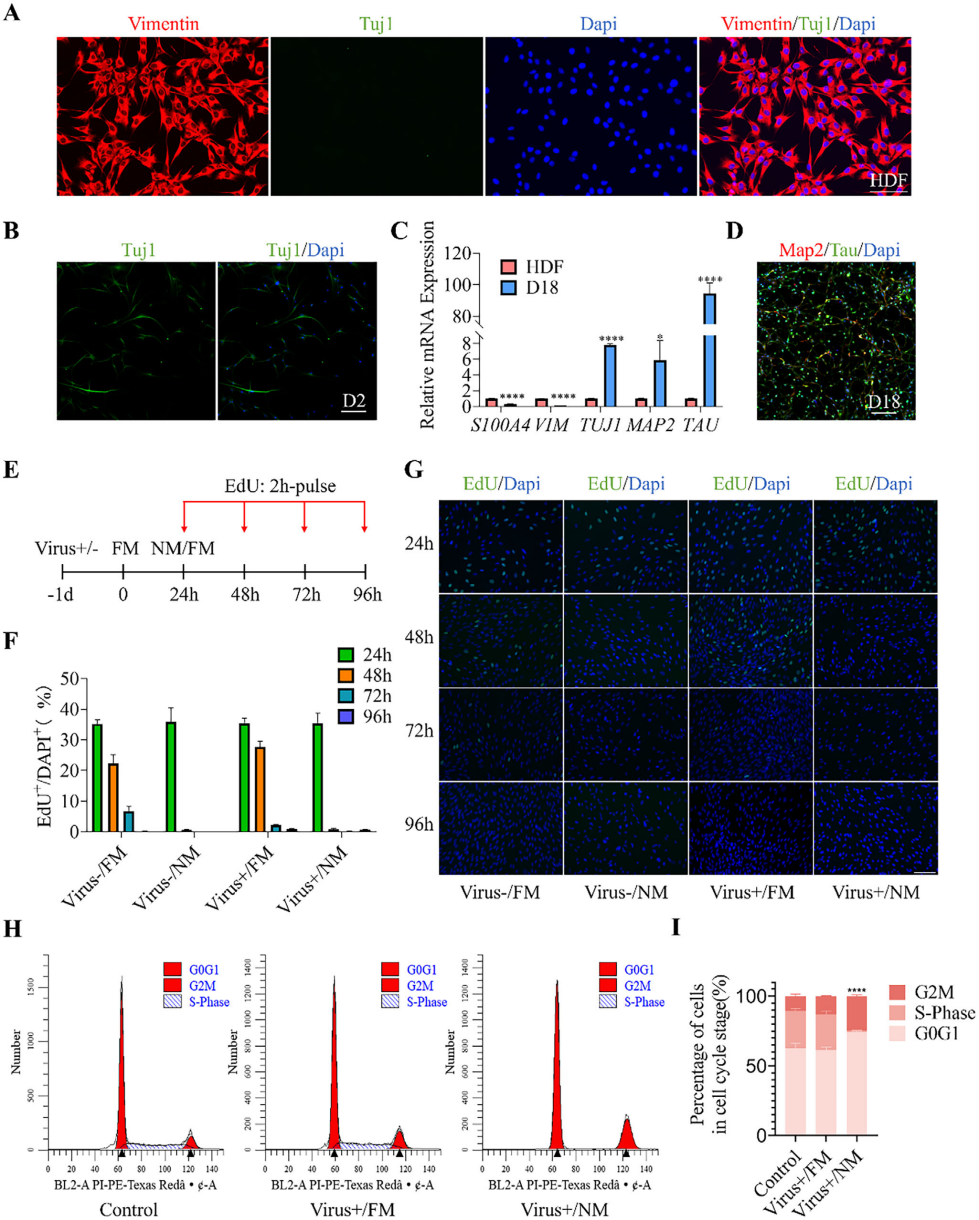
Generation of human induced neurons. A) Immunofluorescence staining of the fibroblast marker Vimentin (red) and neuronal marker Tuj1 (green) in HDF cells. Scale bar: 100 µm. B) Immunofluorescence staining of the neuronal marker Tuj1 (green) in cells on day 2 of transdifferentiation. Scale bar: 100 µm. C) Real‐time qPCR quantification of fibroblast‐associated genes (*S100A4*, *VIM*) and neuronal‐associated genes (*TUJ1*, *MAP2*, *TAU*) at day 18 of transdifferentiation. GAPDH and human fibroblasts were used as internal and negative controls, respectively. *p < 0.05, ***p < 0.001, ****p < 0.0001 compared to HDF. D) Immunofluorescence staining of neuronal marker proteins Tau (green) and Map2 (red) in cells at day 18 of transdifferentiation. Scale bar: 100 µm. E) Experimental timeline for EdU pulse‐labeling experiments in (F) and (G). Cells were incubated with EdU for 2 h at the indicated times. FM: fibroblast medium; NM: neuronal induction medium. F) Quantification of the percentage of EdU⁺/DAPI⁺ cells at 24, 48, 72, and 96 h during direct reprogramming. G) Representative images of cells co‐stained for EdU (green)/DAPI (blue) at 24, 48, 72, and 96 h. Scale bar: 100 µm. H,I) Cell cycle distribution determined by flow cytometry at 48 h. H) Cell cycle flow cytometry diagram of cells under different processing conditions. I) Quantification of the proportion of cells in G_0_/G_1_, S, and G_2_/M phases based on cell cycle analysis. ****p < 0.0001.

To investigate the morphological transformation associated with fibroblast‐to‐neuron transdifferentiation, we initiated lineage conversion via direct reprogramming, as detailed in the Experimental section. **Figure** **1**A presents the overall morphology of in vitro cultures derived from human dermal fibroblasts (HDF). Prior to differentiation, we confirmed that fibroblasts express the marker protein vimentin but do not express the neuronal marker—tubulin Tuj1. Following the introduction of neuronal medium, cells exhibited Tuj1 positivity after 24 h (Figure 1B). On Day 18, quantitative polymerase chain reaction (qPCR) analysis revealed a marked decrease in fibroblast‐specific gene expression (Figure 1C), accompanied by a significant upregulation of most standard neuron markers including Map2 and Tau (Figure 1D). These results indicate the success of direct reprogramming of fibroblast cells to pre‐mature neuron cells.

Noteworthy, we observed minimal cellular proliferation during the transdifferentiation process, as evidenced by 5‐ethynyl‐2ʹ‐deoxyuridine (EdU) incorporation assays. Cells were pulse‐labeled with EdU (10 µm) for 2 h and subsequently imaged at 24, 48, 72, and 96 h following viral infection (Figure 1E). At the 24 h time point, ≈35% of the cells were EdU‐labeled, regardless of viral transfection status (Figure 1F,G). After 48 h, ≈30% of cells treated with fibroblast medium (FM) retained the EdU label, whereas less than 2% of cells treated with neuronal induction medium (NM) were EdU‐positive. By 72 and 96 h, virtually no cells treated with NM were EdU‐labeled, indicating that NM in conjunction with small molecules facilitated cell cycle exit within 24 h. Furthermore, cell cycle analysis demonstrated that the NM culture condition significantly reduced the proportion of cells in the S‐phase (Figure 1H,I). Collectively, these findings suggest that the HDF cells are directly and efficiently reprogrammed into neurons without undergoing a progenitor stage.

To investigate the morphological phenotypic alterations occurring during the transdifferentiation process, we utilize fluorescent labeled fixed‐cell imaging to generate snapshot imaging datasets comprising Dio (3,3ʹ‐dioctadecyloxacarbocyanine perchlorate)‐labeled phase‐contrast microscopy images. Fixed‐cell imaging facilitates the quantification of a substantial number of cells at a discrete time point, thereby minimizing the confounding effects of cellular movement and sample degradation. This method enhances the statistical power necessary for comparing cellular phenotypes across varying experimental condition.^[^ ^20^ ^]^ Moreover, fixed‐cell assays are relatively straightforward to execute, employing fluorescent staining and imaging techniques that permit extended observational periods. These assays enable the concurrent staining of cells with multiple markers, thereby elucidating specific cellular components and providing a comprehensive phenotypic overview. Given that the majority of morphological changes occur during the initial phase of transdifferentiation, we acquire images throughout the first 10 days of the fibroblast‐to‐neuron conversion process. Specifically, once the transduction with the lentiviral vector (designated as Day 0, D0) was completed, the cells were subjected to daily fixation, staining, and imaging procedures until Day 10 to obtain the morphological changes indicative of the transdifferentiation process. Subsequently, the acquired images were annotated to delineate cellular contours using the Labelme software, facilitating the analysis of cellular shape transformations.

### 2.2 Reconstruction of Force Field from Sparse Sampled Single‐Cell Morphological Data

Numerous attempts have been undertaken to quantify the morphological changes in cell populations in response to stimuli, such as exposure to chemical compounds,^[^ ^6^ ^]^ carcinogenesis,^[^ ^7^ ^]^ or cellular reprogramming.^[^ ^21^ ^]^ Current methodologies frequently culminate at the stage of trajectory inference,^[^ ^12^ ^]^ lacking a comprehensive measure of global stability. Although certain studies have highlighted variations in cell shape mode distributions,^[^ ^11^ ^]^ they fall short of developing a dynamic model that elucidates the underlying mechanisms. The quasi‐potential landscape approach,^[^ ^16^ ^]^ reliant on prior knowledge, provides an incomplete and potentially inaccurate representation due to its inherent simplicity and the complex requirement of estimating numerous kinetic parameters. Despite these endeavors, accurately quantifying the underlying morphological dynamics remains a formidable challenge. However, a thorough and precise understanding of the mechanisms driving changes in cell morphology necessitates a deeper insight into the underlying nonequilibrium dynamics.

**Figure 2:**
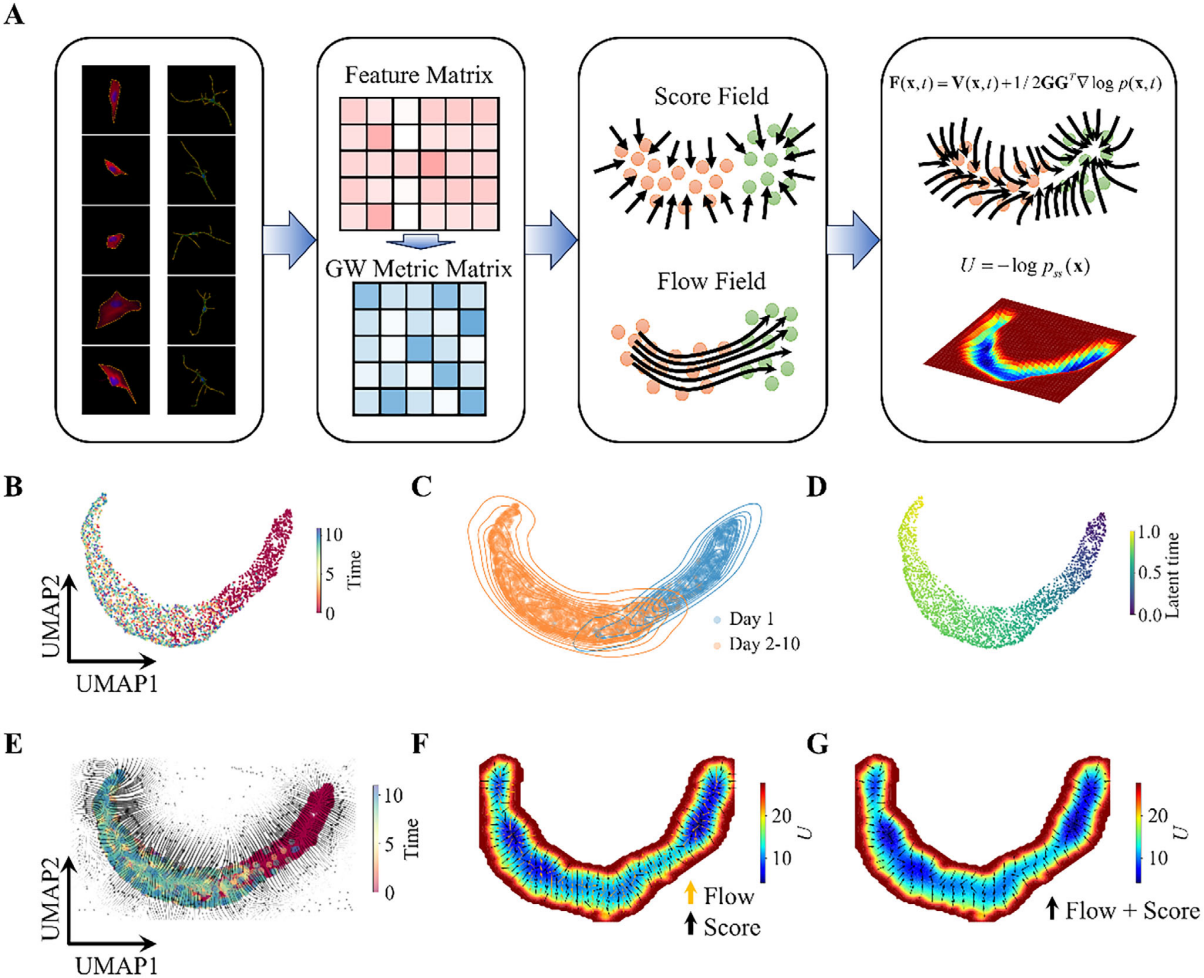
Quantifying morphological landscape with force field reconstruction. A) Illustration of the sparseFFR method. B) The morphological landscape of individual cells was visualized by projecting the data into a two‐dimensional UMAP space, with recording times indicated as continuous colors. C) Estimation of state density according to the UMAP projection. Two major states were identified according to the silhouette analysis on k‐means clustering. D) Latent time was inferred using the pseudo‐diffusion method under the restriction of optimal transport. E) The inferred force field as shown by the streamlines, line width indicates the intensity of the force. F) The morphological landscape obtained by simulating the Langevin dynamics with *D*  =  0.003, and the normalized site‐specific curl flux and gradient force shown by orange and black arrows. Initial states were randomly sampled from the empirical data distribution of Day 1. G) The site‐specific force shown as black arrows on the morphological landscape. In both (F) and (G), the morphological landscape outside the attractive basins are omitted for better visualization and the color scale denotes the landscape potential *U*  =  ‐log *p*_ss_.

In this work, to elucidate the mechanisms underlying fibroblast‐to‐neuron transdifferentiation from a morphological standpoint, we utilize the framework of nonequilibrium statistical mechanics, modeling the morphological transformation as a stochastic dynamical system. The driving force field can be decomposed as described in Equation (6), we endeavor to reconstruct the force field from sparse sampled data by deriving cell‐specific flow and estimating the instantaneous probability density of states. The overarching methodology, which we termed sparseFFR, is depicted in **Figure** **2**A. Initially, we perform segmentation on the microscopic cell images to extract the shape masks $\left\{C_{i}\right\}i=1,\text{…},N_{C}$. Subsequently, cells are centered and aligned along their principal axis using a rotation matrix, along with the sampling of a sufficient quantity of points equidistantly distributed along each contour, typically numbering 150 points. The collection of coordinates $q={\left\{q_{k}^{x},q_{k}^{y}\right\}}_{k=1,\text{…},150}$ of these sampled points are stacked to construct a feature matrix (***X***)_*i*_ =  ***q*** (*C_i_*), yielding a 300‐dimensional state space, $x\;\in \;R^{300}$. The inherent sparsity in the high‐dimensional morphological state space poses a challenge to information‐theoretic margins, constraining the rate at which force field information can be parsed from the data set.^[^ ^22^ ^]^ To accurately reconstruct the force field, we propose projecting both the high‐dimensional state space and the force field into a lower‐dimensional space. Nonetheless, arbitrary projection can distort the probability density, compromising the dependability of force field inference. Preserving its structure after projection is therefore essential for faithfully recovering the underlying dynamics. To circumvent this, we apply the Uniform Manifold Approximation and Projection (UMAP) technique to derive a low‐dimensional representation that preserves the topological structure, as measured by cross‐entropy.^[^ ^23^ ^]^ The resultant projected coordinates form a composite of nonlinear basis functions denoted as {σ_α_(·)}_α  =  1, …, *d*′_, where α specifies the dimension of the reduced representation (α = 2 in this study). The high‐dimensional force field can thus be robustly estimated by ***F***  =  ***Hf***, where ***f***  =  **σ**(***F***) represents the driving force in the reduced state space, and *H* is the projection coefficient matrix. This approximation mirrors a least‐squares fit of the force field through the linear combination of nonlinear basis functions. Despite using a reduced‐dimensionality force field, it retains the capability to recover the nonequilibrium dynamics of the cell cycle.^[^ ^24^ ^]^ The UMAP algorithm demands an estimation of state space metrics. Considering that disparities in cell morphology are invariant to rigid spatial transformations, we compute the Gromov–Wasserstein (GW) distance, a computationally efficient proxy for the Gromov–Hausdorff distance, across sampled cell pairs.^[^ ^13^ ^]^ The GW distances establish a correspondence between distributional pairwise distances of sampled points within each cell, thus forming a metric matrix. This pairwise metric matrix facilitates embedding the sampled cells into a low‐dimensional space. Embeddings of the sampled cells are illustrated in Figure 2B.

The challenge of deducing the flow field from single‐cell morphological data lies in determining both the direction and magnitude of cell‐specific flow. Optimal transport theory emerges as a promising mathematical framework for addressing this issue, as it seeks to minimize the transportation cost between two distributions. This approach has been widely applied to elucidate trajectories in cellular dynamics.^[^ ^25^, ^26^ ^]^ Nevertheless, relying exclusively on optimal transport between temporally ordered snapshots can neglect the within‐time‐point dynamics, which are particularly significant when snapshots are separated by considerable time intervals.^[^ ^27^ ^]^ To overcome this limitation, we integrate optimal transport across successive time points with a cell pair transition matrix to infer the effective probabilities of state transitions, similar to the RealTimeKernel in CellRank 2.^[^ ^27^ ^]^ The complete transition matrix is then constructed as a block matrix (see Methods). The diagonal blocks represent the transition probabilities within a single time point, known as intra‐time‐point transition probabilities, while the off‐diagonal blocks represent the transition probabilities between different time points, referred to as inter‐time‐point transition probabilities. Deterministic local cell‐specific flows on the reduced morphological state space are then estimated by calculating the first‐order Kramers–Moyal coefficient,^[^ ^28^ ^]^ considering both intercellular distances and temporal intervals:

(1) $$\begin{array}{cc} V\;\left(x_{i}\right) & =\lim_{\Delta \tau \to 0}\frac{1}{\Delta \tau }\int p\left(x_{i},\tau +\Delta \tau |x_{i},\tau \right)\;\;dx\\ & \approx \sum_{x_{j}\in N\left(x_{i}\right)}{\hat{T}}_{\mathrm{ij}}\left(\frac{x_{j}-x_{i}}{\tau \left(x_{j}\right)-\tau \left(x_{i}\right)}\right) \end{array}$$

where $N(x_{i})$ is the collection of the nearest neighbors of $x_{i}$. The derived cell‐wise flow field is depicted in Figure S1A (Supporting Information).

Meanwhile, we estimate instantaneous state probability densities using a Gaussian mixture model (GMM), which integrates *N* Gaussian distributions of equal weight, each corresponding to a sample point. This setting corresponds to model with the least prior knowledge following the maximum entropy principle. The sample points’ positions are designated as the means of their respective Gaussian distributions, with a scaled identity covariance matrix **Σ**  =  σ ***I***, where σ is a constant standard deviation optimized to maximize sample likelihood under smoothness constraints on the mixture density. The state density is thus obtained by summing the weighted Gaussian distributions, reflecting the relative population, as illustrated in Figure 2B, and the cell‐wise score field is presented in Figure S1B (Supporting Information). Here, the score field refers to the gradient field of the time‐dependent log‐probability distribution, ***S *** =  ∇log *p*(***x***, *t*), which captures the local directional tendency of state transitions. Finally, cell‐wise driving forces are computed by integrating the flow field ***V*** with the score field ***S*** via a constant diffusion parameter ***G***, as detailed in Equation (6).

To facilitate simulation and further analysis, we employed regularized kernel methods as a tool for obtaining a smoothness‐constrained global force field across the reduced morphological state space, as shown in Figure 2E.^[^ ^17^, ^29^ ^]^ This smooth global force field is encapsulated as a parameterized function, capable of faithfully representing a high‐dimensional force field, as learned through the expectation‐maximization (EM) algorithm.

Overall, our sparseFFR methodology enables the reconstruction of the global driving force field (***F*** (***x***, *t*), Equation (6), utilizing sparse sampled single‐cell morphological data. Since ***F*** (***x***, *t*) is determined across the entire space, exhaustive simulation of the system's Langevin dynamics becomes possible. Notably, through an independent parameterization of the flow and score fields, sparseFFR supports the simulation of stochastic processes with arbitrary diffusion coefficients. This capability has demonstrated critical importance for elucidating the dynamics and thermodynamics of the morphological changes of fibroblast‐to‐neuron transdifferentiation, as illustrated in the subsequent discussions.

### 2.3 Quantifying the Morphological Landscape and Identification of Cell Shape Modes

Employing our sparseFFR methodology, we can represent the global force field as a continuous and analytic function, enabling, for the first time, the recovery of a morphological landscape defined by the steady‐state probability density function *U*  =  ‐ log *p_ss_*. The inferred morphological landscape is depicted in Figure 2F,G, derived from simulations of Langevin dynamics defined by the force field and a spatially homogeneous diffusion tensor. Notably, inferring this morphological landscape requires no prior knowledge beyond the sparse sampled cell contours. The congruence between the data points and the inferred morphological landscape is illustrated in Figure S2 (Supporting Information), demonstrating that the morphological landscape accurately reflects the distribution of the sampled data points. This morphological landscape facilitates a comprehensive characterization and stability assessment of the system, as described in previous studies.^[^ ^30^ ^]^ In addition, the normalized site‐specific curl flux ***V**_ss_* = ***J**_ss_* /*p_ss_* and the landscape gradient *U* at steady state are presented in the Figure 2F. The curl flux characterizes the infinitesimal rotational dynamics of a cell state within the force field, devoid of gradients, and represents the driving force between different phases, while the landscape gradient delineates the direction and magnitude of forces stabilizing macroscopic phases. The site‐specific driving forces [Equation (6)], depicted in Figure 2G, are a composite of the curl flux and the landscape gradient. When the driving force from the flux surpasses the landscape gradient, the cell traverses the barrier, facilitating downstream morphological changes. Biologically, this suggests that the cell has accrued sufficient energy from epigenetic transformation and nutrient supply to undergo a significant alteration in shape, indicative of a phase transition. Conversely, if the driving force is insufficient, the cell remains in its initial phase, suggesting it is not yet primed for substantial cytoskeletal reformation.^[^ ^31^ ^]^

**Figure 3:**
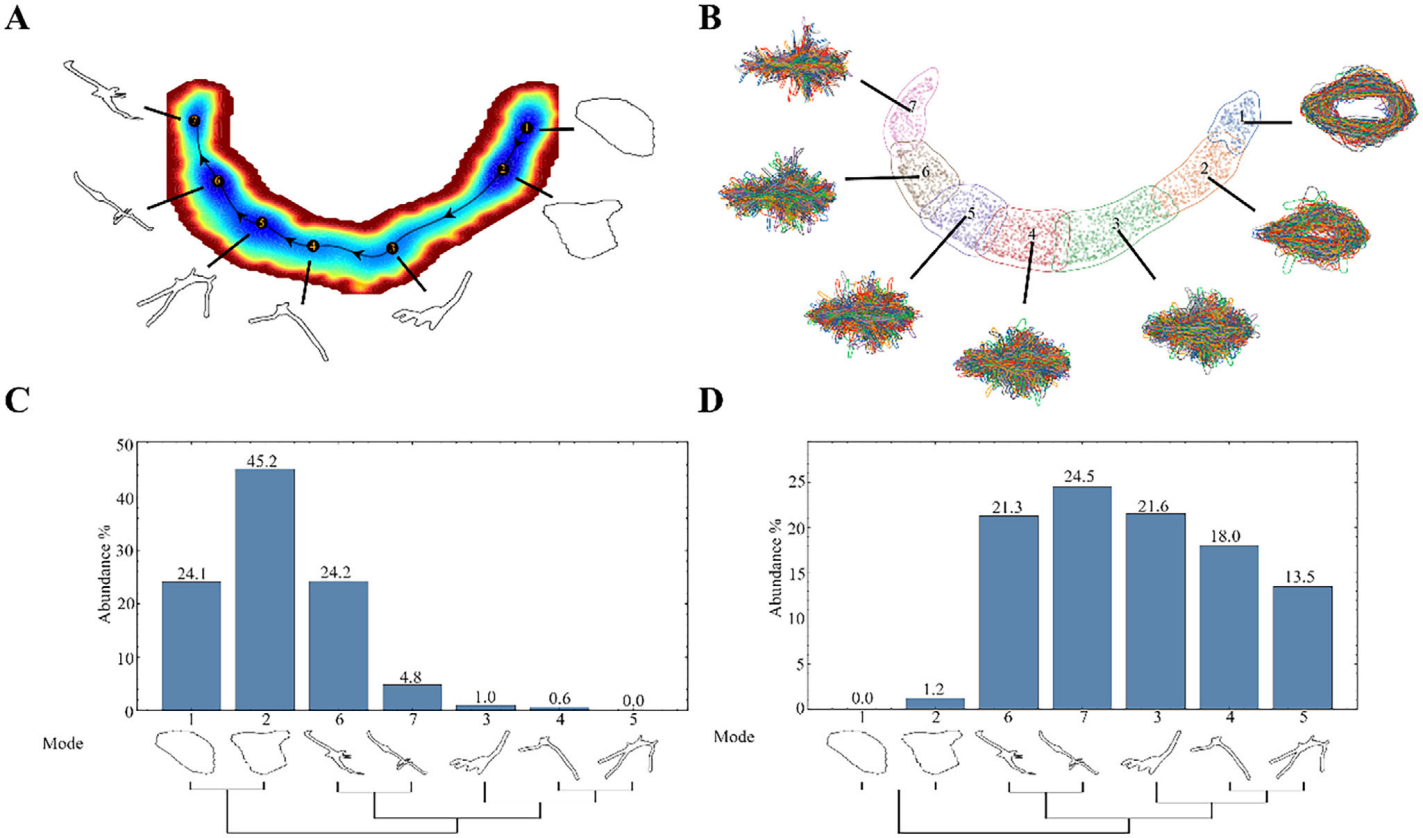
Least action path and distribution of shape modes. A) Least action path computed by finding the most probable path between cell state fixed points. The cell shape mode contour corresponding to each fixed point was reconstructed from the intracellular distance matrix using multidimensional scaling. B) Characterization basins on the morphological space using the k‐means clustering, the basin boundaries of fixed points are indicated by solid lines, and the collection of realigned shape contours are also shown corresponding to each basin. Boundaries of basins are defined using the GMM with *p*(*x*|*c*)  >  0.5 similar to ref. [16] C,D) Shape mode distribution for macrostate 1 and macrostate 2, respectively. The shape mode contours were drawn beneath each vertical bar to indicate the appearance of that shape mode. Arrangement of clustering were indicated with dendrogram to illustrate the topological relationship of the basins on the morphological landscape.

To elucidate the morphological landscape and visualize variations in cellular morphology, we identified the local basins within the landscape. As illustrated in **Figure** **3**A, seven distinct local basins correspond to seven distinct archetypal cellular shapes. For a biological interpretation of these fixed‐point attractors, we propose considering the local basins as cluster centers for characterizing cell shape modes. Cellular clustering was conducted using k‐means clustering, wherein basin locations were fixed as cluster centroids, as depicted in Figure 3B. Compared to shape modes derived from contour alignment and Leiden clustering (refer to Figure S3, Supporting Information), employing basins explicitly delineates the various stages of the phase transition process. Notably, the contour alignment method erroneously classified fluctuations around a basin as separate shape modes, complicating the analysis with additional clusters and obscuring mode distribution at the saddle point of the morphological landscape. In contrast, the distribution of shape modes determined relative to the local basins within the morphological landscape reveals a clear demarcation of seven clusters along the least action path, demonstrating distinct transformations between these two phases (Figure 3C,D). The dendrogram depicting the hierarchical relationship of cell shape modes suggests a two‐phase clustering scenario. Furthermore, unsupervised clustering algorithms alone may perform inadequately on irregular shape clusters or exhibit sensitivity to clustering hyperparameter.^[^ ^11^ ^]^ Employing the local basins of the morphological landscape to inform clustering mitigates these issues, enabling robust and consistent identification of cell shape modes and a reasonable relationship of shape modes.

**Figure 4:**
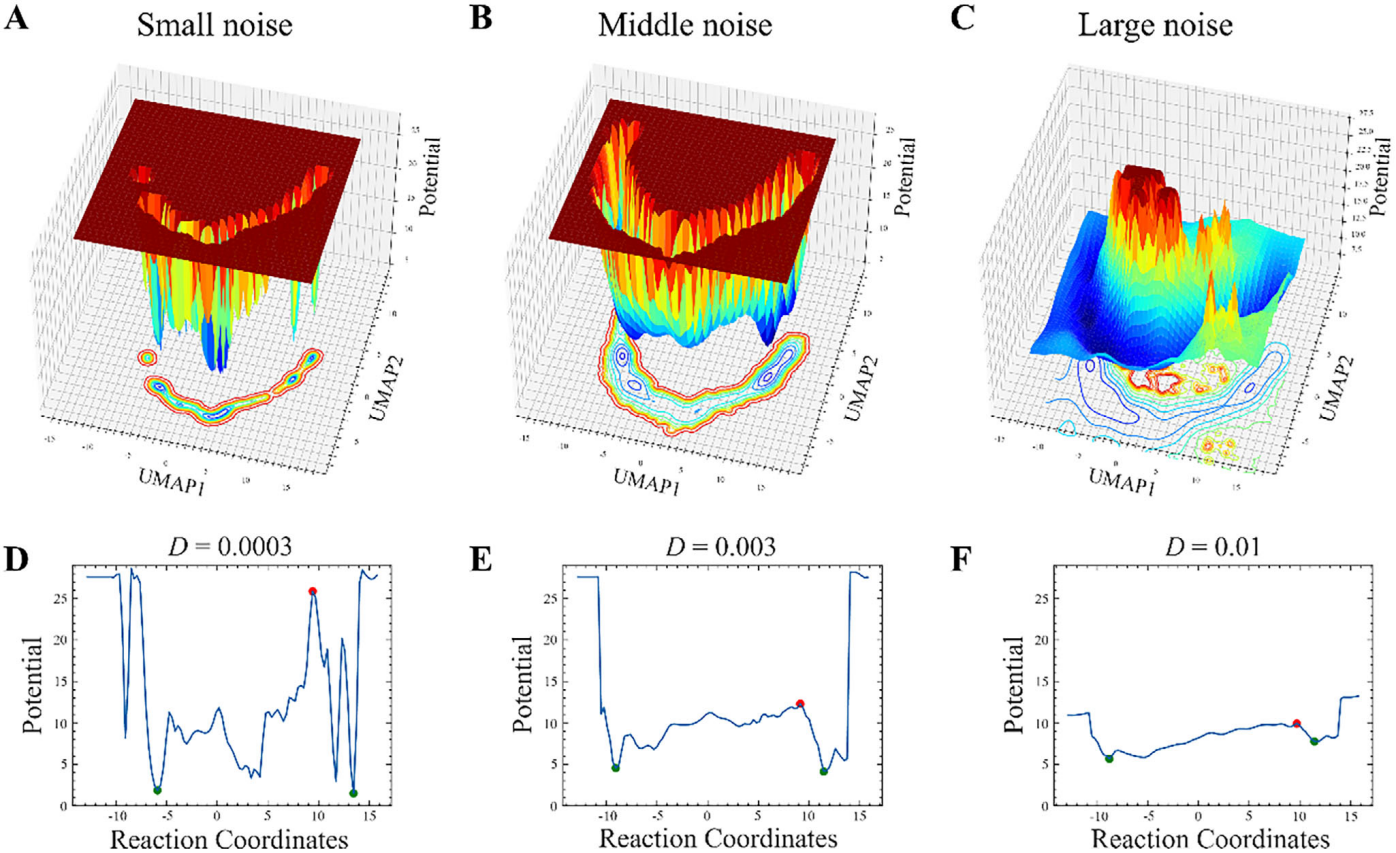
Influence of noise intensity on the morphological landscape. A–C) The 3D morphological landscape and 2D contours are shown for small (*D*  =  0.0003), middle (*D*  =  0.003), and large noise (*D*  =  0.01). D–F) Dependency of landscape potential on the reaction coordinates, location of transition barrier and state stable basins are shown with red and green dots, respectively.

The computation of the least action path as in Equation (12) within a stochastic framework offers a powerful methodology for elucidating the dynamics of complex systems (**Figure** **4** and Methods). The optimal path between successive local basins is identified by varying the continuous trajectory that connects the source basin to the target basin, concurrently updating the transition time. This optimal path defines a deterministic trajectory analogous to that of a deterministic system governed by the variational principle. Consequently, a reduced model can be identified that captures the essential dynamics of the system by focusing on the most significant transition pathways. This model approximates the behavior of the original system but with significantly fewer degrees of freedom, thereby allowing for more efficient analysis and simulation of the system's behavior. In essence, the path of least action can be regarded as the reaction coordinate,^[^ ^32^ ^]^ given that the majority of system trajectories adhere to this path from reactants (fibroblasts) to products (neurons). This approach is particularly useful in high‐dimensional systems where direct simulation of the full model would be computationally prohibitive. The insights derived from this reduced model not only deepen our comprehension of stochastic dynamical systems but also facilitate intuitive and precise predictions of the system's behavior in the absence of noise, as elaborated in subsequent discussions.

### 2.4 Global Dynamics and Thermodynamics under Variant Noise Intensities

The derivation of a parameterized global force field facilitates the simulation of stochastic dynamics [Equation (2)] of the system across a spectrum of noise intensities. This approach permits a comprehensive examination of how noise influences the fundamental mechanisms driving morphological transformations during transdifferentiation, through the lens of nonequilibrium dynamics and thermodynamics.

Critically, by conceptualizing the least action path as the reaction coordinate, we achieve a reduced‐dimensionality representation of the intricate transdifferentiation process, thereby elucidating its intrinsic two‐phase mechanism.

Figure 4 illustrates the significant impact of noise strength on the morphological landscape. In the regime of low noise (Figure 4A,D), distinct local basins are demarcated by substantial energy barriers, indicating a propensity for the system to remain in the fibroblast state for extended durations. Consequently, transdifferentiation is driven by infrequent, large fluctuations can surmount these barriers. The morphological landscape along the reaction coordinate is characterized by considerable roughness, necessitating its interpretation as a multiphase dynamical system, given the prolonged entrapment of the system in each local basin. As noise intensity escalates to an intermediate level (Figure 4B,E), a two‐phase scenario emerges, featuring two prominent basins separated by a substantial global barrier. Each of these major basins encompasses many smaller local energy minima, functioning as extended dynamical attractors and representing regions of phenotypic resilience and stability. Accordingly, we designate these two prominent basins as emerged macrostates, characterized by stable cellular morphology and distinct morphological features. This dynamic definition of macrostates aligns well with clustering analyses of cell shape modes (Figure 3C,D), underscoring the self‐consistency of our method between dynamical and phenotypic dimensions. With the large global basins identified as macrostates, the smaller local basins are identified as mesostates, representing mesoscopic local stable clusters. Transitions between mesostates occur with greater frequency than those between macrostates, introducing a timescale separation, a phenomenon observed in numerous physical and biological systems.^[^ ^33^ ^]^ In the high noise regime (Figure 4C,F), the two macrostates persist, albeit with diminished mesostates. This persistence of macrostates suggests the system can be described consistently as a two‐phase system separated by a finite, high global barrier, analogous to protein folding dynamics.^[^ ^34^ ^]^

**Figure 5:**
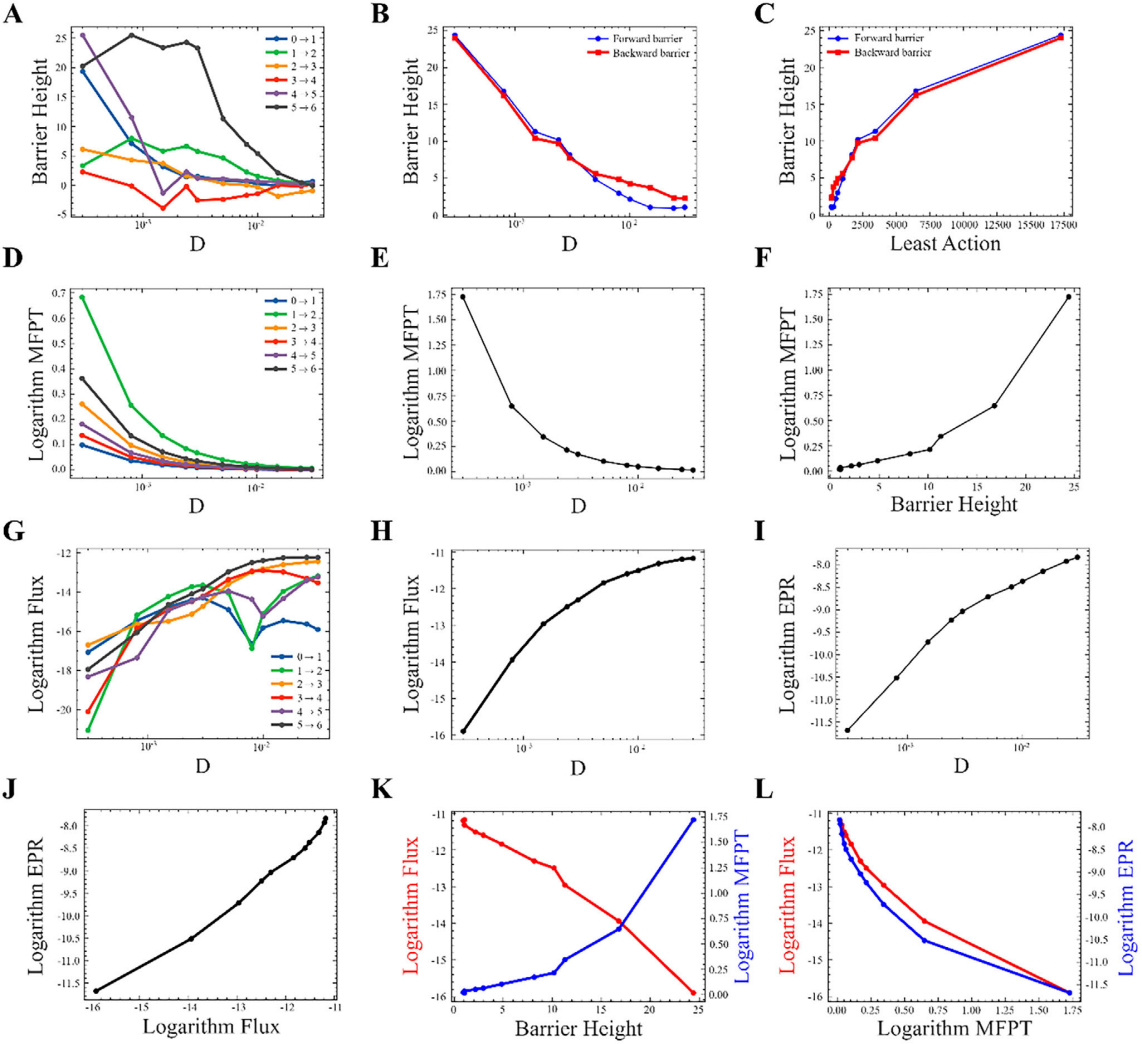
Thermodynamic relations inferred from the morphological landscape. A,B) Dependency of local and global barrier heights on diffusion coefficient *D*. C) The correlation between global barrier height and least action along most probable path when diffusion coefficient is changed. D,E) Impacts of various diffusion coefficients on local and global MFPT. F) Relationship between global barrier height and global MFPT. G,H) Correlation of individual and global integral flux with diffusion coefficient. I) Dependency of EPR on diffusion coefficient. J) Correlation of global integral flux to EPR. K) Relationship of global barrier height with global integral flux and global MFPT. L) Relationship of global MFPT with global integral flux and EPR.

The first‐order phase transition between these two macrostates is expected to be governed by Arrhenius‐like processes, wherein the transition probability exhibits an exponential dependence on the barrier height and is inversely proportional to the noise intensity. To quantify this phase transition and substantiate the aforementioned hypothesis, we examined the relationship between the morphological landscape and the diffusion coefficient D. As illustrated in **Figure** **5**A, there exists a negative correlation between the barrier height and the diffusion coefficient across all small local basins. This results in an almost linear relationship between the global forward and backward barriers over a substantial range of diffusion coefficients, as depicted in Figure 5B. In addition, we analyzed the dependency of the forward and backward barrier heights on the least action along the most probable path. Figure 5C demonstrates a clear increase in barrier height with respect to the least action. In other words, an increase in noise intensity leads to a reduction of the barrier height, thereby requires smaller action. To further quantify the difficulty of escaping local or global attractors, as measured by the mean first‐passage time (MFPT), we present the dependency of MFPT on local and global barrier crossings in Figure 5D,E. The MFPT exhibits a pronounced negative dependency on diffusion noise, indicating that an increase in noise intensity results in a significant reduction in dwell time within both local and global basins. This observation aligns with the reduction of small barriers separating the local basins.

With increasing noise intensity, the MFPT exhibits a progressive decline. As depicted in Figure 5F, there is an approximately linear positive correlation between the global barrier height and the logarithm of the MFPT in the low‐barrier region; however, this correlation intensifies significantly at higher barrier heights. This observation suggests that noise effectively diminishes the least action required for transdifferentiation, thereby lowering the potential barrier and subsequently reducing the MFPT. The stability of the macrostate correlates positively with the barrier height, necessitating greater action to initiate the phase transition from fibroblast to neuron. To elucidate the nature of the driving force behind this phase transition, we explore the relationship between the accumulated flux and noise intensity [Equation (13) and Methods]. As indicated in Figure 5G, the logarithm of the accumulated flux demonstrates a positive proportionality to noise intensity, suggesting that increasing fluctuations correspond to an enhanced probability flux.

This trend becomes more pronounced when considering the global accumulated flux, as illustrated in Figure 5H. An increase in flux enhances the driving force, thereby facilitating transitions between adjacent local basins. Consequently, there is a heightened frequency of barrier crossings, manifested as an elevated probability at these barriers, ultimately resulting in a reduction of the barrier height *U*  =  ‐log *p*_ss_ at steady state. To elucidate the underlying mechanisms of flux augmentation and associated nonequilibrium thermodynamics, we investigate the dependency of the entropy production rate (EPR) on noise intensity [Equation (15) and Methods]. As depicted in Figure 5I, increasing noise intensity contributes to greater entropy production. The net effect is a positive correlation between flux and entropy production (Figure 5J), indicating that an increase in flux demands a stronger thermodynamic dissipation. The close interplay of barrier height with flux and MFPT is depicted in Figure 5K, where an elevated barrier height is associated with a longer MFPT and diminished flux. Consequently, an elongated MFPT emerges as a direct consequence of reduced flux, corresponding to smaller EPR, as shown in Figure 5L. From a thermodynamic viewpoint, noise can be considered as a source of driving force perturbing a system's stability; large noise intensity promotes more extensive exploration of the state space, thereby contributing to increased entropy and subsequently elevating the entropy production rate. In other words, the more dissipation, the easier to cross the transition barrier. From this viewpoint, we can treat noise as an additional thermodynamic force, beyond gene regulation, facilitating cell phenotype conversion.

In conclusion, noise plays a crucial role in the morphological transformation during fibroblast‐to‐neuron transdifferentiation. Elevated noise levels lead to an increased flux, thereby enhancing the likelihood of state delocalization, which in turn accelerates morphological transitions between shape modes and reduces the escape time necessary to initiate transdifferentiation. Conversely, the gradient of the landscape tends to stabilize the current state by maintaining it within the present attractor. A higher barrier on the landscape further stabilizes states, resulting in prolonged switching times and consequently inefficient phenotype transitions. However, the augmented driving force from noise incurs the cost of increased thermodynamic dissipation, as indicated by a higher rate of entropy production. Our findings therefore highlight a fundamental trade‐off between thermodynamic dissipation and the efficiency of cell fate switching. Cells must balance the advantages of noise‐induced barrier reduction against the drawbacks of increased dissipation consumption and potential overheating during transdifferentiation. Elucidating how cells optimize this balance by leveraging the beneficial aspects of noise while minimizing its costs can provide deeper insights into their remarkable efficiency in achieving functionality with limited thermodynamic expenditure.

### 2.5 Identification of Morphology‐Related Driver Genes

**Figure 6:**
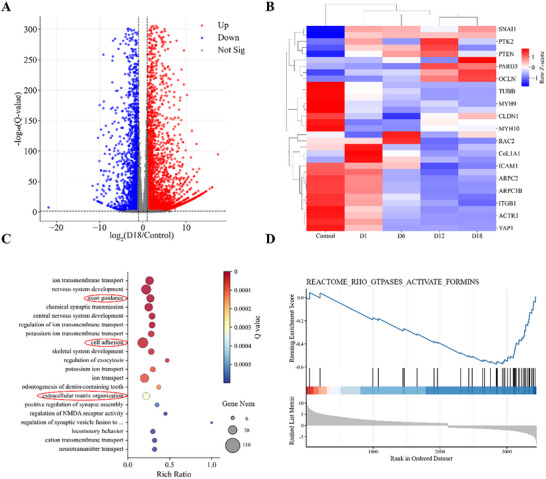
RNA sequencing analysis and the heatmap of related genes during transdifferentiation. A) Volcano plot of changes in gene expression in HDF and induced neurons at Day 18. Upregulated and downregulated genes are shown using red and green dots, respectively. No differentially expressed genes (no‐DEGs) are indicated as gray dots. B) Heatmap of morphology‐related genes during transdifferentiation (identified from Gene Ontology (GO) knowledge base). Signal intensity is based on normalized counts per million reads mapped (FPKM) values, and data are shown as a Z‐score‐normalized log_2_FPKM. The red represents values above the mean, while white represents the mean, and blue represents values below the mean. Three independently repeated samples were collected for each test. C) GO biological process enrichment analysis of genes upregulated at Day 1 and Day 6. GO analysis was performed using the *phyper* function in R. D) The GSEA plot illustrating the enrichment of the ECM proteoglycans pathway from the msigdb_c2_cp_reactome database. The *y*‐axis represents the Enrichment Score (ES), which quantifies the degree to which a particular gene set is overrepresented at the top or bottom of the ranked gene list. The *x*‐axis corresponds to the ranked gene list, with the most significantly differentially expressed genes at the extremes.

In order to explore the morphology‐related gene changes during transdifferentiation, bulk RNA sequencing was employed to assess differential gene expression. The resulting volcano plot (**Figure** **6**A) illustrates the comprehensive alterations in gene expression throughout the transdifferentiation process. Compared to control cells, there is a pronounced alteration in gene expression patterns, with ≈3731 genes upregulated and around 2491 genes downregulated. These shifts in gene expression are largely attributable to changes in cellular functionality. Given that direct reprogramming is utilized to induce fibroblast‐to‐neuron transdifferentiation, it is anticipated that morphological changes in cell shape are also regulated by gene expression alterations. Consequently, we conducted a detailed analysis of the expression changes in key morphology‐related genes from Day 0 to Day 18, as depicted in the heatmap (Figure 6B). This analysis reveals that numerous genes associated with cell shape, structure, and tissue organization exhibit markedly different expression levels during transdifferentiation. On Day 0, two clusters of morphological genes are highly expressed. The first cluster comprises genes that facilitate cell shape maintenance, mobility, and division, including *CDK1, TUBB, TUBB4B, MYH9, CDC42, CLDN1, MAPK1*, and *MYH10*. The second cluster encompasses genes with analogous functions, such as *ARPC2, VIM, ARPC1B, ACTG1, ITGB1, ACTB, ACTR3, RAC1*, and *YAP1*, which are involved in cytoskeletal structuring and reorganization, thereby supporting cell integrity, shape, and stability. Shortly after reprogramming, on Day 1, a distinct group of genes associated with muscle contraction and the tensile strength of connective tissues, including *MYL9, COL1A1*, and *COL1A2*, are upregulated. Concurrently, genes responsible for maintaining cell shape are down‐regulated, indicating that the cells are poised to undergo morphological changes and participate in tissue formation. Following this phase, genes related to neuronal cell shape, such as *TUBA1A*, and actin cytoskeleton regulation, such as *RAC2*, begin to be upregulated, suggesting a surge morphological transition from fibroblasts to neurons. By Day 18, additional neuron‐specific genes are up‐regulated, facilitating neuronal functionality. Notably, the alterations in cell shape‐related gene expression predominantly occur during the early stages of transdifferentiation, characterized by a surge in expression pattern changes. This observation aligns with our morphological landscape model, which predicts a macroscopic two‐phase dynamic process delineated by a transition barrier.

To clarify the major events characterizing the morphology‐shift phase (Day 1–6) of cellular reprogramming, we conducted a comprehensive functional profiling of biological processes. The Gene Ontology (GO) terms that indicate of the top enriched annotation clusters are depicted in Figure 6C. Relative to Day 1 cells, marked upregulation of pathways such as axon guidance, cell adhesion, and extracellular matrix (ECM) organization was observed during the transdifferentiation process. As is well‐established, activation of the axon guidance pathway is instrumental for neuronal outgrowth and neural network formation. The enhanced expression of axon guidance genes implies an establishment of the molecular groundwork necessary for neural connectivity and synaptogenesis, correlating with significant morphological transformations observed in cells throughout the initial stages of transdifferentiation. In contrast, downregulated genes were predominantly associated with cell division pathways (Figure S4A, Supporting Information). Moreover, Gene Set Enrichment Analysis (GSEA) of RNA sequencing data disclosed an enrichment in the Rho GTPases activate formins and other cell cycle‐related pathways (refer to Figure 6D and Figure S4B, Supporting Information), suggesting its downregulation in this biological context. Given the critical role of Rho GTPases in cell adhesion, migration, and signaling, the down‐regulation of the Rho GTPases activate formins pathway indicates active cytoskeletal remodeling during the morphology‐shift stage. Collectively, the regulation of these pathways highlights that fibroblasts undergo extensive cytoskeletal architecture reconfiguration and neural guidance modifications during the morphology‐shift phase, bolstering the acquisition of neuron‐like characteristics and potential interconnectivity. This supports the molecular and structural transition toward a neuron‐like phenotype and function. A recent investigation underscored the feasibility of inducing human foreskin fibroblast‐to‐neuron conversion through cytoskeletal disruption.^[^ ^35^ ^]^ Evidence suggests that molecule inhibitor‐mediated cytoskeletal disruption can dismantle the original lineage expression profile, prompting an intermediate state. The intricate dynamics of the reprogramming trajectory, alongside the pivotal role of cytoskeletal remodeling in facilitating transdifferentiation and phenotype conversion, are consistent with our findings. This suggests that the remodeling of the cytoskeleton may constitute a pivotal obstacle in the process of cellular reprogramming. The destabilization of cytoskeletal integrity appears to amplify stochastic fluctuations, thereby aiding the traversal of the transition barrier.

### 2.6 Method Validation on Public Datasets

To benchmark sparseFFR, we analyzed a recently released public dataset from Copperman et al.^[^ ^12^ ^]^ This dataset comprises 48 h of live‐cell imaging of MCF10A cells subjected to EGF stimulation, sampled every 30 min. Following the methodology, we reconstruct the morphological landscape *U*  =  ‐log *p*_SS_ and compare it with the UMAP embedding of single‐cell measurements (Figure S5I, Supporting Information). The morphological landscape presents a comprehensive characterization and stability assessment of the system. The inferred global force field enables the deduction of key thermodynamic properties, as demonstrated in Figure 5.

To assess the applicability of sparseFFR to additional single‐cell modalities, including scRNA‐seq, we analyzed the dataset reported by Zhu et al.^[^ ^24^ ^]^ The dataset comprises spliced and unspliced single‐cell transcriptomes from U2OS‐FUCCI cells processed via the Snakemake pipeline (https://github.com/CellProfiling/FucciSingleCellSeqPipeline). Comparing the RNA‐velocity‐based inference with the similarity‐only approach reveals a clear vertex‐like progression ordered as M → M‐G1 → G1‐S → S → G2‐M, with G0 positioned outside the ring, consistent with exit from G1 and entry into a quiescent state. The cell‐cycle assignments are further supported by the probability landscape *U* (Figure S6H, Supporting Information), which exhibits a Mexican‐hat topology across the cycling phases and a distinct basin corresponding to the resting state outside the cycle.

The detailed procedures for the methods tested on the two datasets are provided in the Supporting Information. It is noteworthy that prior work, exemplified by the dynamo algorithm,^[^ ^29^ ^]^ constructed a global force field f(x) solely from cell‐wise velocities. In this formulation, f(x) encodes the locally averaged collective velocity over y∈ε(x) as a deterministic vector, and cell‐state dynamics are modeled by the Langevin equation $\frac{dx}{dt}=f\left(x\right)+\xi \left(t\right)$, where **ξ**(*t*) is Gaussian, state‐independent, and satisfies 〈**ξ**(*t*)〉 ≡ 0. Identifying f(x) as the force field is justified insofar as $\left\langle \frac{dx}{dt}\right\rangle =f\left(x\right)$, with the ensemble average 〈 · 〉 taken over all admissible velocities at fixed *x*. However, single‐cell measurements typically provide only one sampled velocity at a given state ***x***, rendering the substitution of f(x) for the true force field a conflation of spatial averaging 〈 · 〉_***x***_ with velocity averaging 〈 · 〉_***v***_.

By contrast, for the general Langevin system $\frac{dx}{dt}=F\left(x,t\right)+G\left(x,t\right)\xi \left(t\right)$, the force decomposes as $F\;\left(x,t\right)=V\left(x,t\right)+\frac{1}{2}GG^{T}\nabla \log p\left(x,t\right)$ [Equation (6)], where ***V*** (***x***, *t*) obeys the Liouville dynamics $\frac{dx}{dt}=V\left(x,t\right)$. This Liouville equation is not an ensemble‐averaged relation and therefore does not require sampling the full velocity distribution. Consequently, ***V*** (***x***, *t*) is estimable via local spatial averages 〈 · 〉_**x**_ at each state ***x***. Under homogeneous diffusion, ***F*** (***x***, *t*) is a deterministic, point‐wise vector field that can be recovered by combining the score ∇log *p*(***x***, *t*) with ***V*** (***x***, *t*). Within the potential landscape and flux framework, the dynamo construction corresponds to the zero‐score limit of the true force, thereby eliminating stabilizing intrinsic forces within basins and systematically underestimating transition barriers.

Consequently, both equilibrium and kinetic behaviors are distorted. More specifically, underestimating transition barriers flattens basins, which reduces residence probabilities. The steady‐state probability distribution becomes skewed toward shallower, possibly spurious states. States that should be distinct and well‐separated may blur, leading to overestimated configurational entropy and loss of structural specificity. Moreover, dominant phases may be falsely labeled, causing macro‐state misclassification. From a kinetic perspective, the absence of sufficient intrinsic stability forces inflates transition rates. Since the transition rate depends exponentially on barrier height, even small systematic errors in the height cause exponential overestimation of rates, yielding unphysically fast transitions. Furthermore, slow processes—such as rare configuration switches—will appear frequent, collapsing the hierarchy of timescales and breaking multiscale model validity. As a result, pathways that should be negligible become competitive, leading to incorrect mechanistic inference. In summary, application of the dynamo construction can skew equilibrium populations, collapse timescale separations, inflate transition rates and spurious pathways, and lead to mechanistic misinterpretation and unreliable predictions across models and applications.

## 3 Discussion

Direct cell reprogramming offers a promising approach to generating specific cell types, such as neurons, cardiomyocytes, and pancreatic beta cells, which are otherwise challenging to obtain. This process circumvents the pluripotent state by employing specific transcription factors or molecular cues. The potential applications of direct cell reprogramming are vast, including the generation of patient‐specific cell types for disease modeling, drug screening, and cell‐based therapies. A comprehensive understanding of the mechanisms underlying direct cell reprogramming is essential for advancing the fields of regenerative medicine and therapeutic interventions. ScRNA‐seq data provide a unique opportunity to elucidate the mechanisms of direct cell reprogramming by enabling the inference of cellular dynamics through the concept of RNA velocity.^[^ ^36^ ^]^ RNA velocity serves as a powerful analytical tool, allowing for the inference of the driving force field within a cellular population and facilitating the construction of a landscape that depicts the dynamic behavior and developmental trajectories of cells.^[^ ^29^ ^]^ In contrast, single‐cell morphological data lack inherent temporal information analogous to RNA velocity, as they capture the structural characteristics of cells without conveying directionality or the rate of cellular changes over time. This limitation poses a significant challenge in quantitatively and dynamically describing single‐cell morphological landscapes. Consequently, it represents a major obstacle in the integration and interpretation of single‐cell morphological data with other multi‐omic datasets, thereby hindering our understanding of complex biological systems and the interplay between molecular and structural processes within individual cells.

In addressing the challenge of elucidating the morphological landscape of single‐cell imaging data, we introduce a comprehensive methodology for the reconstruction of the driving force field. Our approach hinges on the decomposition of the driving force field [Equation (6)] into a flow field [Equation (5)] and the score field [Equation (7)] which is the gradient of a potential field. This formula extends the landscape and flux framework prevalent in nonequilibrium dynamics and thermodynamics studies to non‐steady‐state situations. By concurrently estimating the flow flux and score fields, our method circumvents the necessity for computing directional quantities typically required in RNA velocity analyses. This enables the deduction of state‐dependent driving forces governing the stochastic dynamics of morphological transformations during transdifferentiation, utilizing only the sparse sampled single‐cell morphological data. Central to our approach is an accurate depiction of cell‐cell similarity. For studying morphological transitions from fibroblasts to neurons, we employ the Gromov–Hausdorff distance as a transformation‐invariant metric to assess cell‐cell similarity within the high‐dimensional morphological space. Subsequently, we estimate the flow field [Equation (5)], construct a Markov chain based on the similarity matrix, and compute cell‐specific flows via an optimal transportation guided transition probability. Coupled with a score field derived from state density estimated through GMM, the driving force of individual cells can be determined using unsupervised learning. A parameterized, smooth functional form of the driving force field is then attained by fitting a set of basis functions. The morphological landscape, conceptualized as the negative logarithm of the steady‐state probability distribution, can thereby be delineated from the driving force field. In summary, our sparseFFR framework provides an efficient and robust strategy for the analysis of single‐cell morphological datasets.

Conventional analyses of cell morphology typically rely on a limited set of geometric descriptors, including area, perimeter, shape factor, and aspect ratio. Widely used pipelines such as CellProfiler and ImageJ/Fiji adopt this paradigm to quantify cellular shape and monitor morphological dynamics.^[^ ^37^, ^38^ ^]^ However, given the intrinsic complexity of cell form, such low‐dimensional representations often fail to capture phenotypic heterogeneity. To better encompass morphological diversity, several approaches pursue high‐dimensional featurization by augmenting geometric and statistical descriptors to construct per‐cell summary vectors. Representative methods include Cell Painting, PhenoPlot, the morphome, cell polymorphism, and CellTraj.^[^ ^8^, ^10^, ^12^, ^39^, ^40^ ^]^

In testing these approaches on fibroblast‐to‐neuron transdifferentiation, we computed 127 quantitative descriptors per cell—49 Zernike moments and 78 shape metrics, consistent with CellProfiler and related pipelines—to assemble a feature vector for each single cell. Aggregating these vectors yielded an observation matrix amenable to dimensionality reduction via PCA (Figure S7A, Supporting Information) and UMAP (Figure S7B, Supporting Information). However, these embeddings did not resolve trajectories consistent with temporal state transitions. More broadly, the feature set failed to capture morphometric differences sufficient to separate cell states, complicating biological interpretation of clusters in the reduced spaces.

To circumvent the limitations of hand‐engineered summaries, several methods directly encode raw cell contours as features, including VAMPIRE and M‐TRACK.^[^ ^11^, ^16^ ^]^ These frameworks approximate complex cellular outlines by sampling a sufficiently dense set of equally spaced points along each contour, thereby generating high‐dimensional coordinate representations. Because these coordinates are sensitive to absolute position and orientation, contours are normalized either by translating centroids to a common origin and rigidly rotating shapes to a predefined major axis (VAMPIRE), or by aligning contours to a mean shape (M‐TRACK). The normalized coordinates constitute the feature vector for each cell, which is then subjected to dimensionality reduction.

To assess the applicability of these methods to highly irregular cell morphologies, such as neurons, we generated normalized cell contours by resampling 150 points along each segmentation boundary and realigning the resulting contours to a mean shape following M‐TRACK. The first 100 contours corresponding to the terminal stage of transdifferentiation are shown in Figure S7C (Supporting Information), with the aligned contours in Figure S7D (Supporting Information). Despite realignment, neuronal shapes remain markedly irregular, and residual variability from orientation and dendritic complexity persists. Consequently, PCA does not fully resolve cells by stage (Figure S7E,F, Supporting Information).

UMAP provides improved stage separation (Figure S7G, Supporting Information). However, relative to UMAP derived from GW distances (Figure S7H, Supporting Information), embeddings from realigned contours fail to capture the macrostate divergence between early and late transdifferentiation. Using identical embedding hyperparameters, the realigned‐contour approach inflates intercell dissimilarities, as reflected by the dispersed distribution of states in the reduced morphology space, likely due to misalignment of late‐stage neuronal shapes.

In subsequent analyses, VAMPIRE applies K‐means or Leiden clustering to identify representative shape modes for a given cell population. However, as shown in Figure S3 (Supporting Information), Leiden‐based modes proliferate markedly, and their interpretability is limited owing to atypical contour alignment. By contrast, we define shape modes as local basins on the steady‐state probability landscape *U*  =  ‐log *p_SS_*. As illustrated in Figure 3, basin‐based modes delineate successive stages of morphological evolution, closely track the least‐action path, and resolve distinct transitions between two macro‐phases.

M‐TRACK adopts a phenomenological model grounded in a priori regulatory‐network knowledge to describe cell dynamics. A principal limitation is the requisite network simplification, which risks obscuring system complexity. Moreover, reliable inference is hindered by high‐dimensional parameter estimation, potentially decoupling model outputs from biological reality. Although such models capture key features of morphological trajectories, they provide limited access to underlying physical mechanisms and are difficult to fit explicitly to experimental observations. In contrast, we infer the effective force field directly by combining score and flow fields within a nonequilibrium thermodynamic framework (Figure 2). The resulting force field exposes the governing physical mechanisms in a reduced state space, enabling direct comparison with empirical data, computation of thermodynamic quantities, and quantitatively accurate dynamical predictions (Figure 5).

In conclusion, leveraging the GW distance to construct low‐dimensional embeddings of cell morphology reveals a causal structure in lineage progression and establishes a principled basis for force‐field reconstruction and thermodynamic analysis. The GW distance operates without predefined morphological descriptors, is invariant to rigid transformations, and scales to arbitrarily complex, heterogeneous morphologies. Building on the morphology manifold derived from GW‐based embeddings, our algorithm recovers the driving forces and thermodynamic landscape along transdifferentiation trajectories with high fidelity. This framework generalizes across diverse cell types with distinct morphologies and integrates seamlessly with complementary single‐cell modalities, enabling multidimensional, multi‐omic profiling of cell‐state transitions.

Within the context of nonequilibrium dynamics and thermodynamics, we explore the dynamics and thermodynamic of the system across varying noise levels. Our findings indicate that increased noise levels substantially affect barrier heights, curl flux, and the coarse‐grained macroscopic mechanisms underlying transdifferentiation. Specifically, elevated noise levels result in reduced barrier heights, thereby decreasing the mean passage time. Furthermore, the curl flux exhibits a monotonic increase with noise level, suggesting an enhancement of process irreversibility, as evidenced by the elevated entropy production rate. These observations imply that random fluctuations act like an additional force, destabilizing the current state and facilitating cell lineage conversion. The augmentation of thermodynamic dissipation intensifies this random force, easing barrier crossing. This observation implies a nuanced interplay between efficiency and thermodynamic costs in the regulation of cellular fate transitions.

As barrier heights diminish, proximate local basins coalesce into a larger basin, representing a macroscopic state throughout the process. This phenomenon exemplifies the emergence of simplicity in macroscopic behavior from complex microscopic dynamics, as discussed by Qian et al.^[^ ^41^ ^]^ Consequently, a macroscopic two‐phase nonlinear dynamics emerges from the underlying stochastic process, with the kinetic rates of this macroscopic dynamic determined entirely by the height of the highest barrier separating the two macroscopic basins. Our results also account for the transition state induced by cytoskeletal architecture disruption through pharmacological perturbation, as described by He et al.^[^ ^35^ ^]^

In conclusion, we introduce sparseFFR, a novel methodology for quantifying the potential landscape utilizing single‐cell imaging datasets devoid of velocity data. Through the application of our method to the analysis of single‐cell imaging data pertaining to fibroblast‐to‐neuron transdifferentiation, we showcase its power in elucidating the dynamics and thermodynamics of cellular behaviors and interactions. Furthermore, our findings unveil a fundamental trade‐off between the efficiency and cost associated with cell fate transitions, potentially indicative of a universal principle governing phenotypic changes across cellular processes. Our approach is readily extendable to other single‐cell multi‐omic datasets, including proteomics, metabolomics, and epigenomics, facilitating the elucidation of the underlying stochastic dynamics and the thermodynamics. Meanwhile, the reliability of the reconstruction is underpinned by several key assumptions. Primarily, adequate sampling across time points on the manifold are generally required. Sufficient temporal coverage is necessary to capture the full spectrum of state transitions; otherwise, the estimated force field may be biased or discontinuous in sparsely sampled regions. Second, the system is assumed to exhibit homogeneous diffusion, with a constant diffusion coefficient throughout the analysis. This simplification is necessary because pseudotime‐resolved single‐cell data lack the resolution needed to robustly infer state‐dependent diffusion, where stochastic fluctuations vary across cellular states. While this assumption is commonly adopted in dynamical reconstructions, it may overlook potential heterogeneity in noise levels, and future approaches that directly capture state‐dependent variability could further refine the inferred dynamics. Third, transitions are approximately memoryless, adhering to the Markov assumption where future states depend solely on the present configuration. Although this assumption facilitates the reconstruction of force fields within the Fokker‐Planck/Langevin framework, it may underestimate potential history‐dependent processes that could regulate cell fate transitions, such as epigenetic memory. These premises collectively define the scope and limitations of the method, establishing that its applicability is contingent upon these conditions of sampling adequacy, dynamical uniformity, and Markovianity. The potentially broad applicability of our method offers a means to quantitatively explore the intricate stochastic characteristics of cellular processes, offering crucial insights that were previous inaccessible.

## 4 Methods

### 4.1 Analysis Overview

Our computational framework reconstructs the potential and flux landscape from single‐cell data through the following integrated workflow: (a) Input contours: Obtaining cell imaging data, segmenting the image, and extracting cell contours. (b) GW/UMAP embedding: computing the GW distance across sampled cell pairs and forming a metric matrix. The data are embedded into a low‐dimensional latent space using UMAP, which preserves the intrinsic geometry of the data. (c) Cell‐wise flow and score field: obtain cell‐wise flow field by computing the first Kramers–Moyal coefficient via transition matrix; and the score field via GMM estimation of the instantaneous state probability density. (d) Global force fitting: Employing regularized kernel methods as a tool for obtaining a smoothness‐constrained global force field. (e) Simulation and landscape/metrics: Obtaining morphological landscape through Langevin simulation of the stochastic trajectory ensemble, which allows computation of landscape metrics and thermodynamics.

### 4.2 Decomposition of Force Field into Flow Field and Potential Gradient

The stochastic dynamics of the cell morphological change are characterized by an overdamped Langevin equation in the morphological state space $x\;\in \;R^{d}$:

(2) $$\dot{x}=F\left(x,t\right)+G\left(x,t\right)\xi \left(t\right)$$

where the vector field $F\left(\cdot ,t\right):R^{d}\mapsto R^{d}$ represents the driving force field, and the diffusion coefficient matrix $G\left(\cdot ,t\right):R^{d}\times R^{d}\mapsto R^{d}$ quantifies the noise intensity. The term ***ξ*** denotes Gaussian noise with the correlation function 〈 ***ξ*** (*t*) ***ξ*** (*t*′)〉 =  δ(*t* − *t*′). The evolution of the marginal probability density *p*(***x***, *t*) is described by the Fokker–Planck equation:

(3) $$\partial_{t}p\left(x,t\right)+\nabla \cdot J\;\left(x,t\right)=0$$

where the probability flux vector $J\;=Fp\left(x,t\right)-\frac{1}{2}\nabla \cdot \left(GG^{T}p\left(x,t\right)\right)$ indicates the rate of probability flow within the state space, as outlined in previous studies.^[^ ^42^ ^]^

The Fokker–Planck equation may alternatively be reformulated as a dynamic transport equation, expressed as

(4) $$\partial_{t}p\left(x,t\right)+\nabla \cdot \;\left(V\left(x,t\right)p\left(x,t\right)\right)=0$$

where the flow field is defined by

(5) $$V\;\left(x,t\right)=F-\frac{1}{2}\nabla \cdot \left(GG^{T}\right)-\frac{1}{2}GG^{T}\nabla \log p\left(x,t\right)$$

This formulation is derived by recognizing that ∇ ·  (***GG*** ^***T***^ *p*(***x***,*t*)) =  *p*(***x***, *t*)∇ · (***GG*** ^***T***^) + *p*(***x***, *t*) ***GG*** ^***T***^∇log *p*(***x***,*t*). Assuming spatial homogeneity in the diffusion, such that ∇ ·  (***GG*** ^***T***^) =  0, the force field can be decomposed as

(6) $$F\;\left(x,t\right)=V\left(x,t\right)+\frac{1}{2}GG^{T}\nabla \log p\left(x,t\right)$$

With time‐dependent score field

(7) $$S\;\left(x,t\right)=\nabla \log p\left(x,t\right)$$

Intuitively, the flux component represents rotational, non‑conservative forces that promote delocalization of states, thereby destabilizing the current configuration and increasing the likelihood of exploring alternative states. In contrast, the landscape gradient represents the deterministic, stabilizing forces that push the system downhill towards potential basins, which are the attractors of the system. This is analogous to the dynamics of a charged particle moving in electric (gradient force) and magnetic (rotational force) field. Equation (4) is formally analogous to a continuity equation of a deterministic fluid dynamical system with the density *p*(***x***, *t*) satisfying Equation (4) whose time evolution of the fluid is governed by the Liouville equation:

(8) $$\dot{\hat{x}}=V\left(\hat{x},t\right)$$

where ***V*** is the flow velocity vector field. Although x and $\hat{x}$ are intrinsically different dynamical systems, the ensemble of trajectories described by Equation (8), initialized from the distribution *p*(***x***, *t*  =  0), reconstructs the marginal probability density as given by Equation (3). This is because the same field function *p*(***x***, *t*) solves both Equations (3) and (4) as probability density distribution and as fluid density, respectively. Consequently, the force field ***F*** is expressed as a combination of a deterministic flow field ***V*** and the gradient of the logarithm of the instantaneous state probability density, denoted as ∇log *p*(***x***, *t*), which we refer to as the score field ***S*** (***x***, *t*).^[^ ^43^ ^]^ At steady state, the flow field is given by ***V***_***ss***_ = ***J***_***ss***_ /*p_ss_*, and the force field is expressed as a combination of a rotational flow flux and a gradient of a potential $F\;=J_{\mathrm{ss}}\;/p_{\mathrm{ss}}-\frac{1}{2}GG^{T}\nabla U$, where ∇ ·  ***V***_***ss***_ =  0 and *U*  =  ‐ log *p_ss_*. This relationship demonstrates that Equation (6) extends the potential landscape and flux theory by considering a time‐dependent potential landscape, which, while not confined to steady‐state, maintains the Helmholtz decomposition structure.^[^ ^44^ ^]^

### 4.3 GW/UMAP Embedding

For the computation of the GW distance,^[^ ^45^ ^]^ we uniformly sampled 150 points along the boundary of each cell segment *i* (yielding 300 coordinates) and computed the pairwise Euclidean distance matrix *D^i^*. The GW distance between cells *i* and *j* is defined as

(9) $$\mathrm{GW}\;\left(D^{i},D^{j}\right)=\frac{1}{2}\;\min_{T^{\mathrm{ij}}\in C}\sum_{\alpha ,\beta ,\delta ,\gamma }\left|D_{\alpha \beta }^{i}-D_{\gamma \delta }^{j}\right|T_{\alpha \gamma }^{\mathrm{ij}}\;T_{\beta \delta }^{\mathrm{ij}}$$

where *T^ij^* denotes a weighted pointwise correspondence between the points of *D^i^* and *D^j^*, and *C* is the set of admissible weighted assignments.

The resulting metric matrix quantifies pairwise similarity between cells *i* and *j*, and serves as a precomputed distance matrix for UMAP. The embedding dimensionality was fixed at 2, with a minimum distance of 0.5 to regulate the compactness of point placement. The parameter n_neighbors=30 was selected to balance local and global structure, and the same neighborhood size was used to construct the neighborhood graph with a corresponding connectivity matrix.

### 4.4 Intra‐ and Inter‐Time‐Point Transition Matrix Inference

We begin by developing a probabilistic framework that allocates transition probabilities to neighboring cells based on morphological similarity, assuming gradual, memoryless transitions along the state space manifold.^[^ ^46^ ^]^ To evaluate the intra‐time‐point transition probabilities, we construct a fuzzy topological graph derived from the k‐nearest neighbor graph, employing GW distances to measure cell similarities.^[^ ^23^ ^]^ This graph represents the high‐dimensional data structure, with edges weighted by the probabilities of connectivity between data points, thereby reflecting their similarity. These edge weights are interpreted as the pairwise transition probabilities, constituting the transition matrix (***T***)_*ij*_ =  *p*(***x***_***i***_| ***x***_***j***_) for cell pairs (*C_i_*,*C_j_*) within a given time point. For inter‐time‐point transition probabilities, we define a transport map $\pi_{t_{i},t_{i+1}}$ for each pair of successive time points *t*_*i*_ and *t*_*i* + 1_, which assigns each cell at *t*_*i*_ to its most likely future state at *t*_*i* + 1_ utilizing Waddington optimal transport (WOT).^[^ ^25^ ^]^ Consequently, the global transition matrix ***T*** is structured as a block matrix, where diagonal blocks represent intra‐time‐point transition probabilities and off‐diagonal blocks represent inter‐time‐point transition probabilities. We then adjust ***T*** by substituting elements in first off‐diagonal block with those derived from the computed transport maps. Normalization of each matrix row yields the optimal transport‐informed transition matrix $\hat{T}$, representing a Markov chain model of the system. Subsequently, we compute real‐time informed pseudo‐time τ(***x***) using a pseudo‐time diffusion method based on $\hat{T}$,^[^ ^25^, ^27^ ^]^ as illustrated in Figure 2D.

### 4.5 Optimal GMM Parameter Selection

As for selecting σ in GMM density estimation, we integrate *N* Gaussian kernels of equal weight, each centered at a sample point, thereby imposing minimal prior structure consistent with the maximum entropy principle. The covariance is set to a scaled identity, **Σ**  =  σ ***I***, where σ is a global bandwidth chosen to maximize sample likelihood under smoothness regularization of the mixture density. In practice, smoothness is quantified by comparing the GMM density to a reference kernel density estimate (KDE) using bandwidth 0.35 (Scott's rule).^[^ ^47^ ^]^ Specifically, the smoothness metric *C*_1_ is defined as the sum of negative KL divergences between the GMM and KDE densities across macrostates, and the log‐likelihood *C*_2_ is the sum of log GMM densities evaluated at all sampled data. We evaluate *C*_1_ and *C*_2_ for σ  ∈  (0.002, 0.2), linearly normalize them to ${\hat{C}}_{1},{\hat{C}}_{2}\in \;\left(0,1\right)$ (Figure S8, Supporting Information), and select σ by maximizing $C={\hat{C}}_{2}-{\hat{C}}_{1}$, yielding an optimum at σ  ≈  0.03. In a Gaussian mixture model, within the range of σ  ∈  (0.002, 0.2), the mixture covers the data points reasonably well. Therefore, the dependency of log‐likelihood on σ is gradual rather than steep, leading to the “plateau” shape of the blue curve in Figure 1. This serves as the selection rule used to pick the best σ for GMM density estimation.

### 4.6 Kernel Regularization Used for the Smooth Force Field

For reconstructing a smooth force field, given *N* sparsely sampled manifold points with tangent vectors $S=\{\left(x_{n},y_{n}\right):n\in N,\in x_{n},y_{n}\in R^{D}\}$, the objective is to learn a mapping *f* that accurately fits the inliers. To accommodate noisy outliers, we adopt a robust estimation framework in which inliers are modeled with Gaussian noise of zero mean and uniform standard deviation σ, while outliers follow a uniform distribution with density 1/*a*, where *a* =  5 denotes the volume of the outlier variation space. The likelihood is formulated as a mixture model,

(10) $$p\;\left(\;y|x\;,\theta \right)=\prod_{n=1}^{N}\left(\frac{\gamma }{2\pi \sigma^{2}}\exp\left(-\beta ∥y_{n}-f\left(x_{n}\right){∥}^{2}\right)+\frac{1-\gamma }{a}\right)\;$$

here γ =  0.9 denotes the inlier proportion and is updated during learning. Force field reconstruction employs Gaussian kernels *k* (*x_i_*,*x_j_*) = exp (− *β*‖*x_i_* − *x_j_* ‖^2^) with an empirical bandwidth $\beta =2/{\left\langle ∥x_{i}-x_{j}{∥}^{2}\right\rangle }_{j\in N_{i}}$, where $N_{i}$ comprises *N*/5 nearest neighbors *j* for each *i*. The noise variance is estimated as $\sigma^{2}=\frac{1}{\mathrm{ND}}\;\sum_{n=1}^{N}∥y_{n}-f\left(x_{n}\right){∥}^{2}$.

We posit the mapping *f* in a vector‐valued reproducing kernel Hilbert space (RKHS) H with reproducing kernel Γ, and impose a Gaussian smoothness constraint $p\left(f\right)\propto \exp\left(-\frac{\lambda }{2}{∥f∥}_{H}^{2}\right)$. The maximum a posteriori estimate (MAP) of *θ* follows from Bayes’ rule as $\theta^{*}=\arg\max_{\theta }\;\;p\left(y|x,\theta \right)\;p\left(f\right)$. We optimize this objective via an iterative EM algorithm using 1000 anchor points, each assigned a Gaussian kernel, with remaining points modeled by uniform distributions. The number of Gaussian kernels is chosen according to dataset size and available computational resources. For datasets with fewer than ≈500 observations (cells), we use the full data for force‐field reconstruction; for larger datasets, we subsample at most 1000 observations for sparse reconstruction. The resulting regularized energy to be minimized is

(11) $$E=\;\frac{1}{2\sigma^{2}}\sum_{n=1}^{N}p_{n}\;∥y_{n}-f\left(x_{n}\right){∥}^{2}+\frac{\lambda }{2}\;{\left\|f\right\|}_{H}^{2}$$

with *λ*  =  3 controlling the balance between data fidelity and smoothness.^[^ ^48^ ^]^

### 4.7 Computation of Reaction Coordinates

This technique is grounded in the variational principles of classical mechanics, where the path of least action minimizes the temporal integral of the Lagrangian. In the small fluctuation limit, as articulated by Freidlin–Wentzell theory,^[^ ^49^ ^]^ the least action path delineates the most probable trajectory for a system transitioning between states or basins. The action functional is expressed as

(12) $$S\;\left[x_{\left(t_{i},t_{j}\right)}\right]=\frac{1}{2}\;\underset{t_{i}}{\int^{t_{j}}}\frac{{\left|\dot{x}\left(t\right)-F\left[x\left(t\right)\right]\right|}^{2}}{D}\mathrm{dt}$$

where the path integral spans the stochastic trajectory linking local basin *i* at time *t_i_* to local basin *j* at time *t_j_*.

### 4.8 Computation of Accumulated Flux Along the Reaction Coordinates

The accumulated flux, denoted as $\hat{J}$, is calculated as the path flux along the least action path and is formalized as

(13) $$\hat{J}=\underset{x_{i}}{\int^{x_{j}}}J\left(x\right)\;\cdot dl$$

At steady state, the flux ***J***_***ss***_ is given by $Fp_{\mathrm{ss}}-\frac{1}{2}GG^{T}\nabla \log p_{\mathrm{ss}}$. Thus, assuming spatially homogeneous noise, the accumulated flux is expressed as $\hat{J}=\underset{x_{i}}{\int^{x_{j}}}F\;p_{\mathrm{ss}}\cdot dl-\frac{1}{2}GG^{T}\left(\log\frac{p_{\mathrm{ss}}\left(x_{j}\right)}{p_{\mathrm{ss}}\left(x_{i}\right)}\right)$.

### 4.9 Computation of Entropy Production Rate

The EPR quantifies the nonequilibrium dissipation of energy and information in open systems, serving as an indicator of the process's irreversibility.^[^ ^50^ ^]^ The entropy of the trajectory ensemble can be expressed as:^[^ ^51^ ^]^

(14) $$S\;\left(t\right)=-k_{B}\int p\left(x,t\right)\log p\left(x,t\right)dx$$

where *k_B_* is the Boltzmann constant, and *p*(***x***, *t*) represents the system's instantaneous probability distribution. Upon differentiation and assuming a constant temperature *T* throughout the process, we derive:

(15) $$T\;\dot{S}=-\int \left(k_{B}T\nabla \log p-F\right)\cdot Jdx-\int F\cdot Jdx$$

In this context, $-\int (k_{B}T\nabla \log p-F)\cdot Jdx$ denotes the EPR, and ∫F·Jdx represents the ensemble‐averaged heat dissipation rate (HDR). At steady state, the ensemble‐averaged change in entropy $\dot{S}$ equals zero, leading to the equivalence of EPR and HDR. Given the typical computational intractability of *p*(***x***, *t*) due to field function integration in high‐dimensional space, we estimate the steady‐state EPR using Langevin simulations in the UMAP‐projected low‐dimensional space. It is important to note that the thermodynamic properties derived from these low‐dimensional representations serve as boundary estimates of the original dynamics, with the entropy production rate representing a lower bound to the total entropy production.

## 5 Experimental Section

### Cell Culture

The 293T cells used in this study were provided by the Chinese Academy of Sciences Cell Bank. The human primary skin fibroblasts were obtained from CTCC (Meisen, Zhejiang, China). The 293T cells were maintained in Dulbecco's Modified Eagle's Medium (DMEM) supplemented with 10% fetal bovine serum (FBS). The HDF cells were maintained in DMEM containing 10% FBS, 1% Non‐Essential Amino Acid solution, 100 units mL^−1^ penicillin and streptomycin. All the cells used in this study were expanded in a humidified 37 °C, 5% CO_2_ incubator. The cells were then dissociated with 0.25% trypsin, spun, and frozen in serum‐free cell cryopreservation solution. Each cell line used in this study was tested regularly for mycoplasma.

### Lentiviral Vectors Production and iNs Generation

The plasmid (U6REST1_U6REST2.hPGK.BRN2.hPGK.Ascl1WPRE) was obtained from Addgene (#101852). 293T cells were seeded into 10 cm dishes. After 24 h, two viral packaging plasmids psPAX2 (6 µg) and pMD2.G (3 µg) together with above‐mentioned plasmid (6 µg) were transfected into 293T cells using Lipofectamine 3000 reagent (Thermo Fisher Scientific). 6–8 h later, the medium was removed and replaced by DMEM containing 10% FBS, and then the cell supernatant containing viruses was harvested at 48 and 72 h after transfection. Supernatants containing viruses were first filtered through a 0.22 µm Millex‐HV Syringe Filter Unit (Merck), then concentrated, and finally stored at −80 °C. The viruses were titrated using a qPCR Lentivirus Titer Kit. All titers were in the range of 1.30 × 10^8^ and 7.04 × 10^8^ units mL^−1^.

Induced neurons were generated with previous protocol.^[^ ^52^ ^]^ Briefly, the fibroblasts were seeded on gelatin‐coated or poly‐l‐ornithine, fibronectin, and laminin‐coated glasses. The fibroblasts were plated at 5 × 10^3^ cells/cm^2^ and infected 1 d later for 24 h with the indicated lentiviruses in the presence of 1 × HiTransG A Viral Infection Enhancer. Cells were then washed three times with PBS and cultured in fibroblasts medium for 24 h. Then medium was changed to neural induction medium (NDiff227; Takara‐Clontech) supplemented with growth factors at the following concentrations: LM‐22A4 (2 × 10^−6^ m, Tocris), GDNF (2 ng mL^−1^, R&D Systems), NT3 (10 ng µL^−1^, R&D Systems) and db‐cAMP (0.5 × 10^−3^ m, Stemcell), and the small molecules CHIR99021 (2 × 10^−6^ m, MedChemExpress), SB‐431542 (10 × 10^−6^ m, MedChemExpress), noggin (0.5 µg mL^−1^, R&D Systems), LDN‐193189 (0.5 × 10^−6^ m, MedChemExpress), as well as valproic acid sodium salt (VPA; 1 × 10^−3^ m, Merck Millipore). Half of the neuronal conversion medium was replaced every 2–3 days. Eighteen days post‐transduction, the small molecules were stopped and the neuronal medium was supplemented with only the growth factors (LM‐22A4, GDNF, NT3, and db‐cAMP) until the end of the experiment.

### Cell Cycle Analysis

The cells were harvested and fixed at 4 °C overnight with 70% ethanol. According to the manufacturer's protocol (Beyotime, China), the fixed cells were washed with PBS and incubated with RNase A and PI for 30 min at room temperature (RT) (25 °C) in the dark and analyzed by flow cytometry (Attune NxT Flow Cytometer, Thermo Fisher). ModFit LT 5.0 software was used to analyze cell cycle distribution.

### Cell Plasma Membrane Staining with DiO

The cells were washed with PBS twice and fixed with 4% PFA for 15 min at RT. Cells were then incubated in 0.1% Triton X‐100 in PBS for 10 min at RT. After washing twice with PBS, incubated with Dye working solution in Cell Plasma Membrane Staining Kit with DiO for 20 min at 37 °C. Then the cells were washed three times with PBS for 5 min each time, followed by the addition of 4ʹ,6‐diamidino‐2ʹ‐phenylindole (DAPI) solution; cells were then incubated for 5 min in the dark, washed three times with PBS, and imaged under an EVOS XL Core Imaging System.

### 5‐Ethynyl‐2ʹ‐deoxyuridine Incorporation

For EdU incorporation detection, 10 × 10^−6^ m EdU were added to the cell culture medium according to the manufacturer's instructions provided in the kit (Beyotime, China). After 2 h, the cells were washed twice with PBS, incubated with 4% paraformaldehyde for 15 min at room temperature, Then they were washed twice with PBS, incubated with 0.3% Triton X‐100 for 10 min, and washed twice with PBS; subsequently, Click Reaction solution was added, and the cells were then incubated in the dark for 30 min and washed three times with PBS for 5 min each time, followed by the addition of DAPI solution; cells were then incubated for 10 min in the dark, washed three times with PBS, and imaged under an EVOS XL Core Imaging System.

### Real‐Time Quantitative PCR

Total RNA was extracted and purified using UNIQ‐10 Column Trizol Total RNA Isolation Kit (Sangon Biotech) according to the manufacturer's instructions. A total number of 15 RNA samples at fixed time point were collected, and then sequenced by DNBSEQ (BGI Tech). After purification, complementary DNA synthesis was achieved by using the PrimeScript 1st StrandcDNA Synthesis Kit (Takara). Real‐time qPCR was performed with TransStart Top Green qPCR SuperMix (TransGen) according to the manufacturer's instructions using the StepOnePlus Real‐Time PCR System (Applied Biosystems) following the procedure: 94 °C for 30 s followed by 40 cycles of 94 °C for 5 s and subsequently 60 °C for 30 s. All of the primers were purchased from Comate Bioscience as shown in Table S1 (Supporting Information). The method for data analysis was 2^−ΔΔCt^ for comparison with the endogenous control glyceraldehyde 3‐phosphate dehydrogenase.

### Immunofluorescence

Cells were washed with PBS twice and fixed with 4% PFA for 15 min at RT. Cells were then incubated in 0.1% Triton X‐100 in PBS for 10 min at RT. After washing twice with PBS, cells were blocked in a solution of PBS containing 2% BSA for 60 min at RT. After that, cells were washed with PBS three times and incubated with primary antibodies diluted with antibody dilution buffer (1% BSA in PBST) overnight at 4 °C. Secondary antibodies (1:500) were added at room temperature in the dark after the cells were washed with PBST three times. After 2 h, cells were washed with PBS and incubated with DAPI staining solution for 5 min. Furthermore, images were captured by an EVOS XL Core Imaging System (Thermo Fisher Scientific) or a Nikon confocal laser scanning microscope (Tokyo, Japan).

### Statistical Analysis

Data are presented as means ± standard deviation (SD) of three independent experiments. All analyses were performed using GraphPad Prism. Independent sample *t*‐tests were used for comparisons between two groups, and one‐way analysis of variance (ANOVA) was used for comparisons among multiple groups. Differences with *P*‐values less than 0.05 were considered statistically significant.

## Conflict of Interest

The authors declare no conflicts of interest.

## Supporting information



Supporting Information

Supporting Information

## Data Availability

The data that support the findings of this study are available in the Supporting Information of this article.
