# Supplementary material for: Quantifying the Single‐Cell Morphological Landscape of Cellular Transdifferentiation through Force Field Reconstruction
Source: Adv Sci (Weinh). 2025 Nov 7;13(1):e12325. doi: 10.1002/advs.202512325 (PMC12767054; doi:10.1002/advs.202512325)
Supplement: Supplementary file 1 — Supporting Information [file ADVS-13-e12325-s001.docx]

Supporting Information

**Quantifying the single-cell morphological landscape of cellular transdifferentiation through force field reconstruction**

*Chudan Yu, Chuanbo Liu^*^, Erkang Wang, Jin Wang^*^*

Methods Applied to Public Datasets:

This section provides the detailed step-by-step procedures for applying the sparseFFR to the two publicly available datasets.

To benchmark sparseFFR, we analysed a recently released public dataset from Copperman et al.^[1]^ This dataset comprises 48 h of live-cell imaging of MCF10A cells subjected to EGF stimulation, sampled every 30 min. Cells were segmented automatically using Cellpose.^[2]^ Representative micrographs and corresponding segmentations are shown at $\text{t}\text{ = 0}$, $\text{t}\text{ = 50}$, and $\text{t}\text{ =}$ 96 in [Figure S5A](#FigureS5).

Single-cell embeddings in the original study were constructed from 92 morphological and texture features (Zernike, texture, shape, and cell-cell metrics), followed by cell tracking to resolve trajectories in feature space. UMAP was then applied to principal components of these trajectory features, either at single time points or by concatenating eight consecutive time points ([Figure 3](#Figure3) of Copperman et al. ^[1]^). We reproduced these embeddings in [Figure S5B-C](#FigureS5): the left panel uses single-time features, whereas the right panel uses eight-time-point trajectory snippets. The blue-to-green path with arrows indicates the direction of motion in embedding space, capturing morphodynamical trajectories associated with cell state transitions; whereas cell density is shown in grayscale.

Following the methodology in the main text, we compute the cell-cell GW distance to embed single-cell morphological profiles. The GW distance is invariant to rigid spatial transformations and aligns distributions by matching pairwise distances among sampled contour points within each cell. As shown in [Figure S5D](#FigureS5), the UMAP embedding derived from the GW geometry provides a more coherent representation of the underlying dynamical process.

We delineate four macrostates via silhouette-guided $\text{k}$-means clustering, and estimate instantaneous state probability densities using a GMM with *N* equally weighted components, each anchored at a sample point ([Figure S5E](#FigureS5)). The resulting density induces the cell-wise score field $\boldsymbol{S}=\nabla\log p\left( x,t \right)$ ([Figure S5F](#FigureS5)).

Conversely, we reconstruct the flow field by estimating intra-time-point and inter-time-point transition matrices via optimal transport across successive time points, followed by local, cell-specific flow estimation using the first-order Kramers-Moyal coefficient ([Equation 1](#eq1) of the manuscript; [Figure S5G](#FigureS5)).

The cell-wise driving forces are obtained by integrating the measured flow with the score field under the landscape-flux decomposition of nonequilibrium dynamics ([Equation 6](#Equation6) of the manuscript). A smoothness-constrained global force field is then inferred via an expectation-maximization algorithm coupled to regularized kernel methods, yielding a parametric representation that captures high-dimensional force structure ([Figure S5H](#FigureS5)).

The resulting force field enables stochastic simulations across diffusion regimes to compute dynamical and thermodynamic observables relevant to morphodynamic state transitions. To assess fidelity, we simulate 600 trajectories, each with $\text{5×1}\text{0}^{\text{5}}$ time steps, to reconstruct the morphological landscape $\text{U}\text{ = -}\log\text{p}_{\text{SS}}$ and compare it with the UMAP embedding of single-cell measurements ([Figure S5I](#FigureS5)).

The morphological landscape presents a comprehensive characterization and stability assessment of the system. Other dynamical and thermodynamics characteristics, such as the location of local basins, the barrier heights, MFPT, the least action path, the line-integrated probability flux, the entropy production rates can all be deduced from the inferred global force field, as demonstrated in [Figure 5](#Figure5) of the manuscript.

To assess the applicability of sparseFFR to additional single-cell modalities, including scRNA-seq, we analyzed the dataset reported by Zhu et al.^[3]^ The dataset comprises spliced and unspliced single-cell transcriptomes from U2OS-FUCCI cells processed via the Snakemake pipeline (https://github.com/CellProfiling/FucciSingleCellSeqPipeline). Single-cell profiles were embedded into a low-dimensional manifold using UMAP, revealing the expected cell-cycle phases ([Figure S6A](#FigureS6)).

We inferred the score field using a GMM-based estimator ([Figure S6B](#FigureS6)). The cell-wise score was computed as the gradient of the instantaneous probability density, $\text{∇}\log\text{p}\text{(}\text{x}\text{,}\text{ }\text{t}\text{)}$ ([Figure S6C](#FigureS6)), and the flow field was obtained from the first-order Kramers—Moyal coefficient ([Figure S6D](#FigureS6)). A globally smooth force field was then estimated via an EM procedure ([Figure S6E](#FigureS6)). The probability landscape $\text{U}$ exhibits at least six local basins in the embedded state space, corresponding to the G0, M-G1, G1-S, S, G2-M, M phases ([Figure S6G](#FigureS6)).

The force-field inference described above does not incorporate velocity information embedded in scRNA-seq data. Given the relatively small sample size ($\text{n}\text{ }\text{=1152}$ cells), the inferred global force field exhibits twisting across phase transitions. Incorporating cell-resolved velocity improves the reconstruction of the force field. Specifically, we use RNA velocity as the cell-wise flow field and obtain a smoothness-constrained global force field by combining the flow and score fields ([Figure S6F](#FigureS6)).

Comparing the RNA-velocity-based inference with the similarity-only approach reveals a clear vertex-like progression ordered as M $\text{→}$ M-G1 $\text{→}$ G1-S $\text{→}$ S $\text{→}$ G2-M, with G0 positioned outside the ring, consistent with exit from G1 and entry into a quiescent state. The cell-cycle assignments are further supported by the probability landscape $\text{U}$ ([Figure S6H](#FigureS6)), which exhibits a Mexican-hat topology across the cycling phases and a distinct basin corresponding to the resting state outside the cycle.

References:

[1] J. Copperman, S. M. Gross, Y. H. Chang, L. M. Heiser, D. M. Zuckerman, *Commun. Biology*, **2023**, *6*, 484.

[2] C. Stringer, T. Wang, M. Michaelos, M. Pachitariu, *Nat. Methods*, **2021**, *18*, 100.

[3] L. Zhu, J. Wang, *Adv. Sci.* **2024**, *11*, e2308879,

**
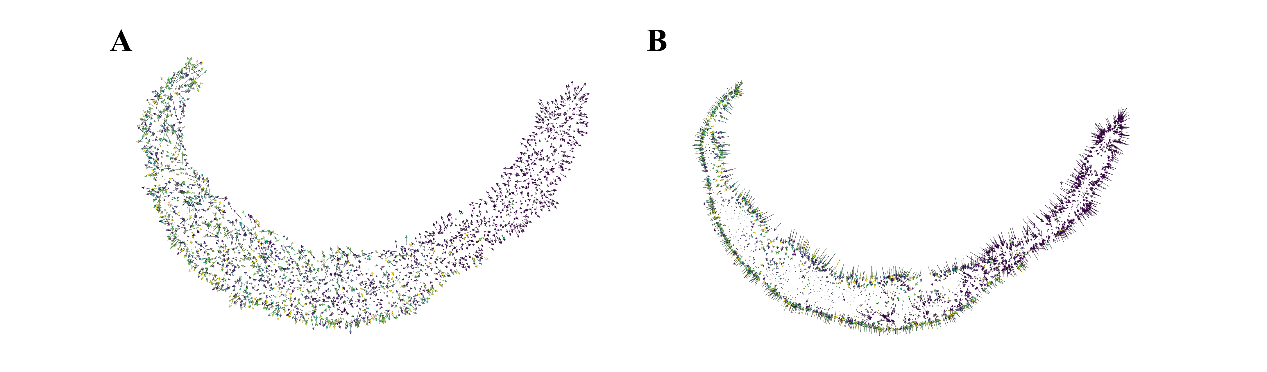
**

**Figure S1.** Cell-wise flow field (A) and score field (B).

**
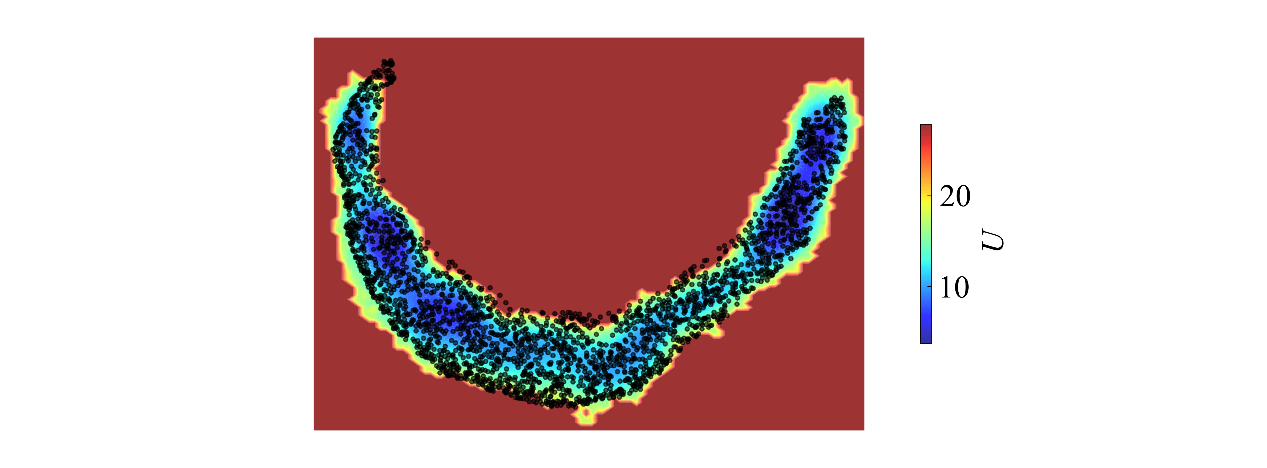
**

**Figure S2.** Overlap of data points with the inferred morphological landscape.

**
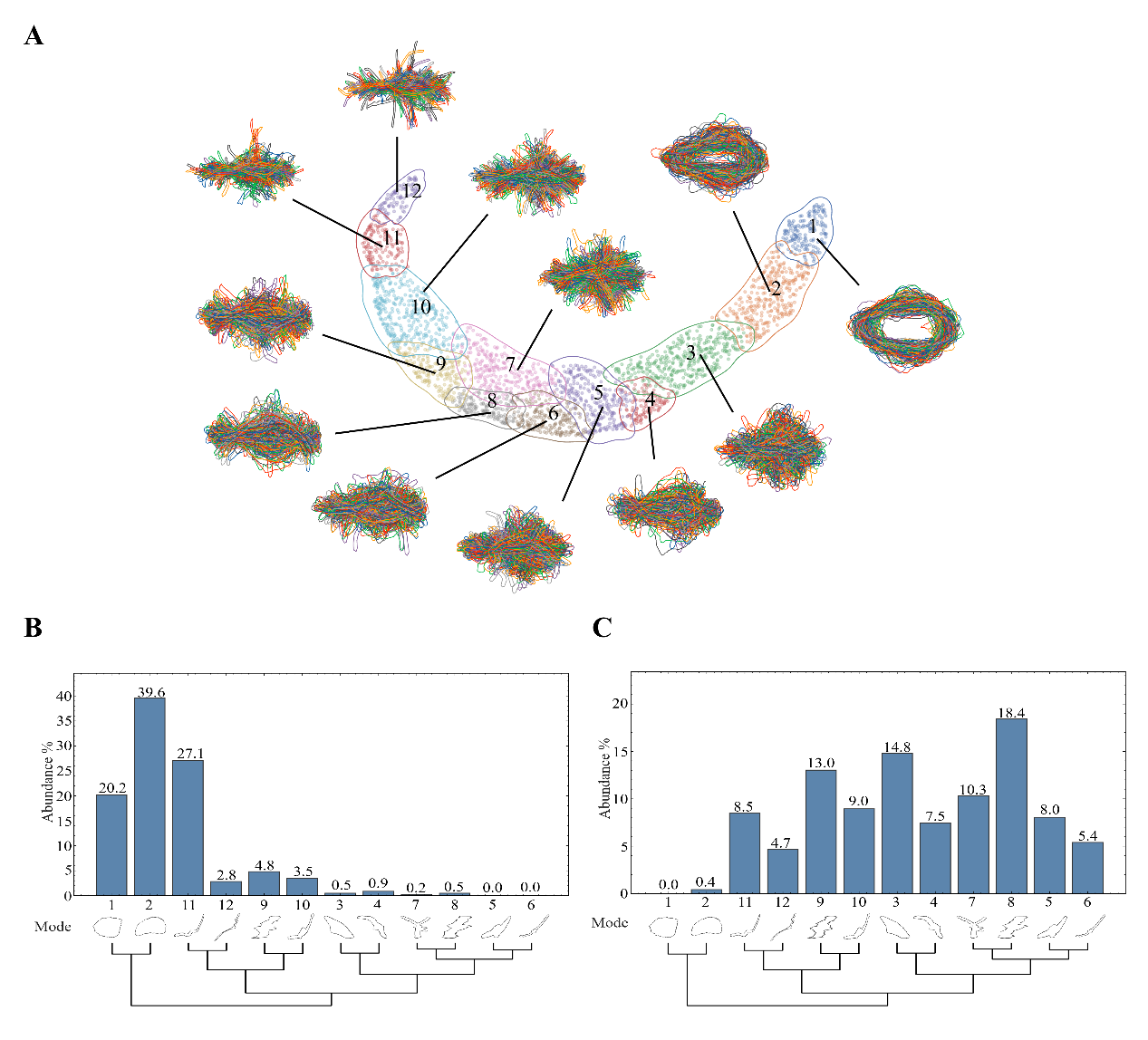
Figure S3.** Shape mode identification based on Leiden clustering. (A) Leiden clustering of aligned cell contours and the contour collection of each cluster. (B) and (C) Bar plots showing the distribution of cell shape modes based on the clustering results before and after the phase transition, respectively. The dendrogram graph bellow the bar plots indicates the hierarchical relation of the shape modes.

**
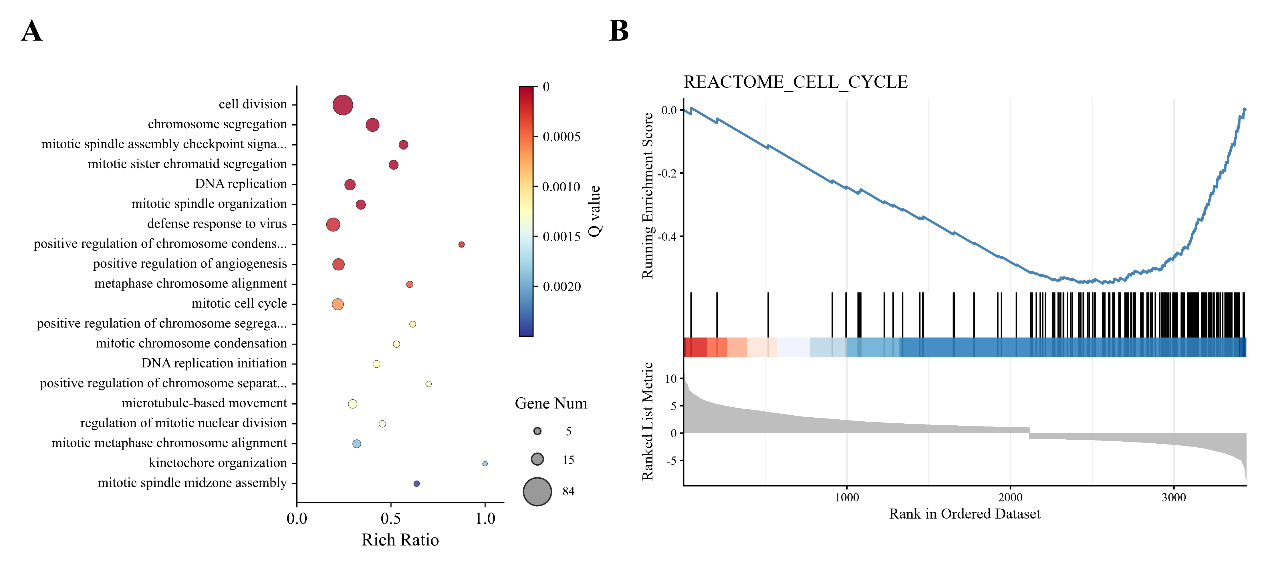
**

**Figure S4.** Enrichment analysis of down-regulated genes at Day 1 and Day 6. (A) GO Biological Process Enrichment Analysis of Genes down-regulated at Day 1 and Day 6. (B) The GSEA plot illustrating the enrichment of the cell cycle pathway from the msigdb_c2cp_reactome database.

**
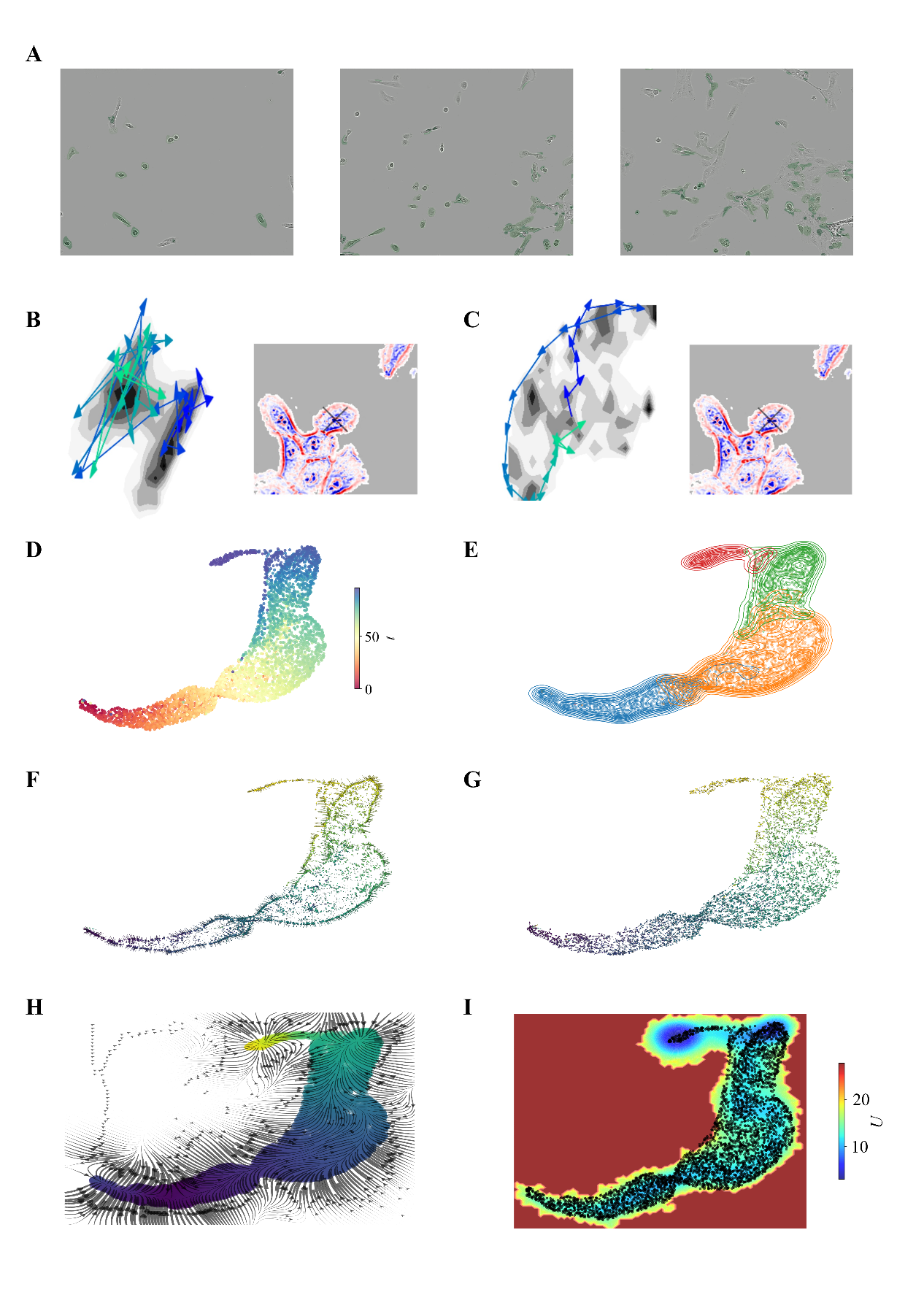
**

**Figure S5.** Application of sparseFFR on public timelapse cell morphology dataset. (A) Microscope images and cell segmentations. (B-C) Cell trajectory embedding using CellTraj with single-time features (B) and 8-time accumulated features (C). (D) Single-cell observation embedding using GW distance and UMAP. (E) Macrostates identified through morphology space embedding. (F) Cell-wise score field obtained by $\nabla\log p\left( x,t \right)$. (G) Cell-wise flow field obtained by computing the first-order Kramers-Moyal coefficient. (H) Global force field obtained through EM algorithm. (I) Probability landscape $\text{U}\text{ = -}\log\text{p}_{\text{SS}}$ obtained through collection of stochastic trajectories with homogeneous diffusion coefficient $D=0.006$ based on the learned force field.

**
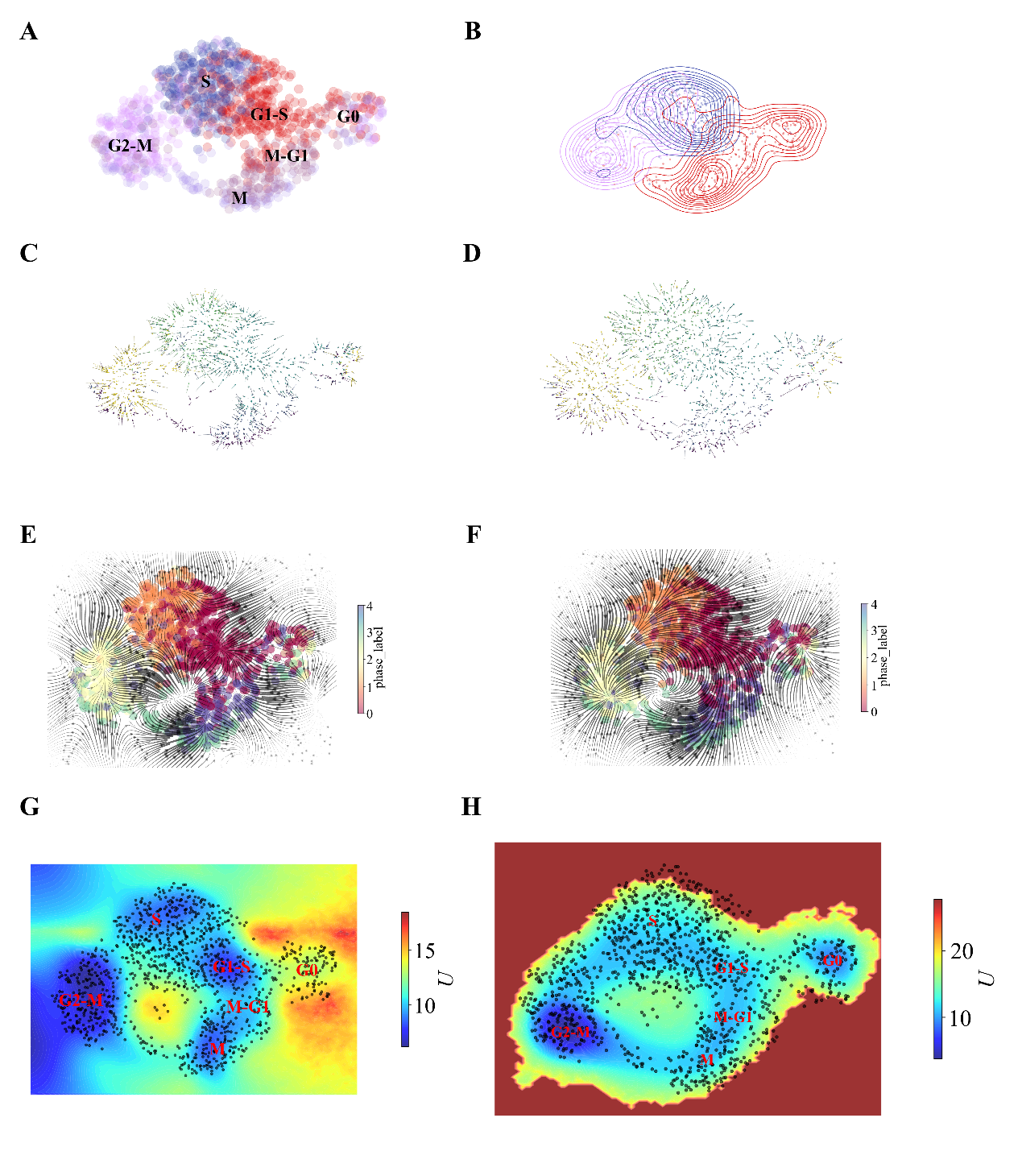
**

**Figure S6**. Application of sparseFFR on scRNA-seq dataset. (A) UMAP embedding of single-cell observations from scRNA-seq of U2OS cells during cell cycle, cell cycle phases are labeled with different colors. (B) We use three macrostates to estimate the instantaneous probability density, corresponding to G2, G1(G0), and S phases. (C) Cell-wise score field obtained from GMM. (D) Cell-wise flow field computed from first-order Kramer-Moyal coefficient. (E) Global force field obtained through combination of score and flow fields. (F) Global force field obtained by incorporating RNA velocity information. (G) Steady-state probability landscape $\text{U}\text{ = -}\log\text{p}_{\text{SS}}$ obtained through collection of stochastic trajectories based on the learned force field. (H) Steady-state probability landscape obtained by using RNA velocity information. The diffusion coefficient used for (G) and (H) is $D=0.006$.

**
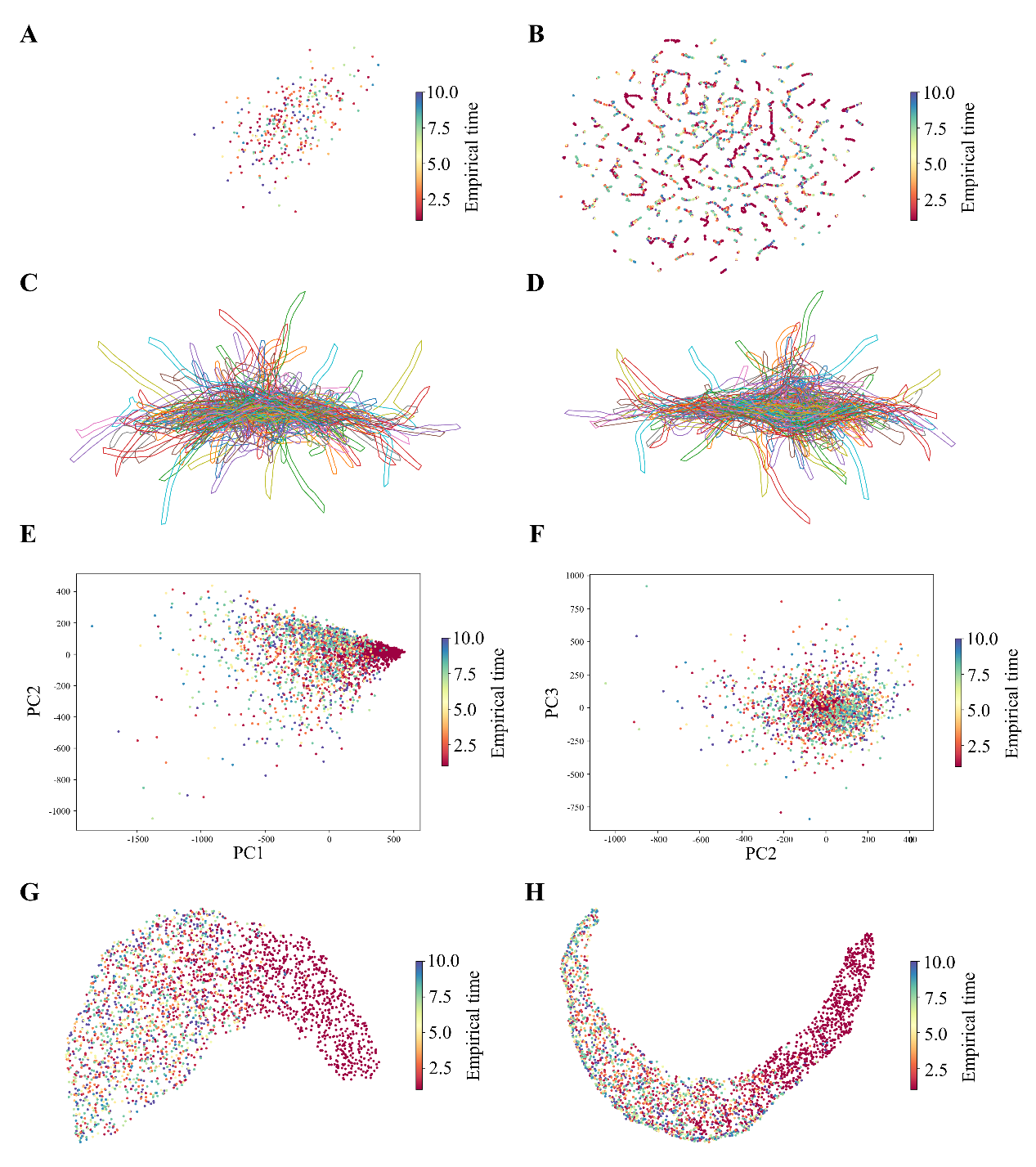
**

**Figure S7.** Comparative study to other cell morphology analysis methods. (A) Embedding of summary features using the first two principal direction of PCA. Both x-axis and y-axis are shown in logarithmic scale to facilitate visualization. (B) Embedding of summary features using UMAP. (C) Representative contours for transdifferentiation of fibroblast cells to neurons. (D) Aligned same set of cell contours as in (C). (E - F) Embedding of single-cell observations on the two leading principal directions ((E) PC1 and PC2), and on the first and third directions ((F) PC1 and PC3). (G-H) Compare UMAP embedding between using aligned contour coordinates and using GW distances. In all embedding figures, the single-cell observations are labeled with empirical time to distinguish the cell state.


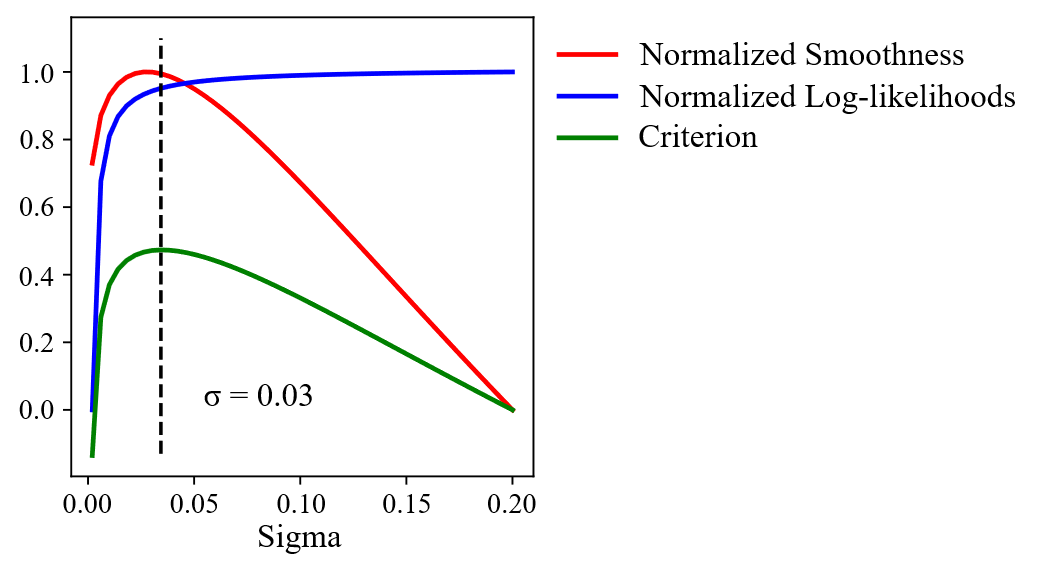


**Figure S8.** Select $\text{σ}$ for probability density estimation with GMM. Normalized smoothness: ${\hat{\text{C}}}_{\text{1}}$; Normalized log-likelihoods: ${\hat{\text{C}}}_{\text{2 }};$Criterion $\text{C}\text{ = }{\hat{\text{C}}}_{\text{2}}\text{-}{\hat{\text{C}}}_{\text{1}}$, balances data fidelity and smoothness to select the optimal $\text{σ}$.

**Table S1.** Primers for qPCR

| Gene | Forward Sequence (5'-3') | Reverse Sequence (5'-3') |
| --- | --- | --- |
| *GAPDH* | ATGTTCGTCATGGGTGTGAA | TGTGGTCATGAGTCCTTCCA |
| *VIM* | AGTCCACTGAGTACCGGAGAC | CATTTCACGCATCTGGCGTTC |
| *S100A4* | GATGAGCAACTTGGACAGCAA | CTGGGCTGCTTATCTGGGAAG |
| *MAP2* | CCCTTTGAGAACACGACACA | TCTGTTAGCGGTGCTGAGGT |
| *TUJ1* | TGGATTCGGTCCTGGATGTG | ACCTTGCTGATGAGCAACGT |
| *TAU* | GAGTCCAGTCGAAGATTGGGT | GGCGAGTCTACCATGTCGATG |
